# Exogenous glutamate rapidly induces the expression of genes involved in metabolism and defense responses in rice roots

**DOI:** 10.1186/s12864-017-3588-7

**Published:** 2017-02-17

**Authors:** Chia-Cheng Kan, Tsui-Yun Chung, Hsin-Yu Wu, Yan-An Juo, Ming-Hsiun Hsieh

**Affiliations:** Institute of Plant and Microbial Biology, Academia Sinica, Taipei, Taiwan

**Keywords:** Rice, Glutamate, Metabolism, Signal transduction, Gene expression, Transcription factor, Defense response

## Abstract

**Background:**

Glutamate is an active amino acid. In addition to protein synthesis and metabolism, increasing evidence indicates that glutamate may also function as a signaling molecule in plants. Still, little is known about the nutritional role of glutamate and genes that are directly regulated by glutamate in rice.

**Results:**

Exogenous glutamate could serve as a nitrogen nutrient to support the growth of rice seedlings, but it was not as effective as ammonium nitrate or glutamine. In nitrogen-starved rice seedlings, glutamate was the most abundant free amino acid and feeding of glutamate rapidly and significantly increased the endogenous levels of glutamine, but not glutamate. These results indicated that glutamate was quickly metabolized and converted to the other nitrogen-containing compounds in rice. Transcriptome analysis revealed that at least 122 genes involved in metabolism, transport, signal transduction, and stress responses in the roots were rapidly induced by 2.5 mM glutamate within 30 min. Many of these genes were also up-regulated by glutamine and ammonium nitrate. Still, we were able to identify some transcription factor, kinase/phosphatase, and elicitor-responsive genes that were specifically or preferentially induced by glutamate.

**Conclusions:**

Glutamate is a functional amino acid that plays important roles in plant nutrition, metabolism, and signal transduction. The rapid and specific induction of transcription factor, kinase/phosphatase and elicitor-responsive genes suggests that glutamate may efficiently amplify its signal and interact with other signaling pathways to regulate metabolism, growth and defense responses in rice.

**Electronic supplementary material:**

The online version of this article (doi:10.1186/s12864-017-3588-7) contains supplementary material, which is available to authorized users.

## Background

Glutamate and glutamine are the first organic nitrogen compounds derived from the assimilation of nitrate and ammonium in plants. In the primary nitrogen assimilation pathway, nitrate taken up from the soil is reduced to nitrite and ammonium by nitrate and nitrite reductase, respectively. Ammonium derived from nitrate or directly absorbed from the soil can be assimilated into glutamine and glutamate via the glutamine synthetase (GS)/glutamine-oxoglutarate aminotransferase (GOGAT) cycle [1–3]. In addition to the primary nitrogen assimilation pathway, glutamate and glutamine can be synthesized via the remobilization of nitrogen-containing compounds and the assimilation of large amounts of ammonium generated by photorespiration in C3 plants [4]. Thus, glutamate and glutamine are closely related in metabolism. Besides glutamine, glutamate can be derived from other amino acids of the glutamate family such as arginine, ornithine, and proline in the plant cell [5].

In addition to protein synthesis, glutamate has many important functions in plants. For instance, glutamate is a major amino donor for the synthesis of amino acids and other nitrogen-containing compounds in plants [5]. The α-amino group of glutamate can be transferred to a wide variety of α-keto acids to form amino acids, which are catalyzed by reversible pyridoxal-5′-phosphate-dependent aminotransferases. In addition to transamination reactions, glutamate can be directly converted to α-ketoglutarate, which is mainly catalyzed by glutamate dehydrogenase (GDH) in plants [5]. The active conversion between glutamate and α-ketoglutarate provides a direct link between nitrogen and carbon metabolism in the cell.

In addition to primary carbon and nitrogen metabolism, glutamate is required for the synthesis of glutathione (GSH), a linear tripeptide of glutamate, cysteine, and glycine and a major intracellular antioxidant in virtually all organisms [6]. Glutamate is also a precursor for the synthesis of photosynthetic pigment chlorophyll. In addition, glutamate can be converted to γ-aminobutyrate (GABA) via glutamate decarboxylase (GDC). GABA is a non-protein amino acid that rapidly accumulates in response to biotic and abiotic stress to modulate plant growth [7–9]. Increasing evidence indicates that GABA may exert its effects in plants through the regulation of carbon metabolism as well as signaling pathways [7–11]. Glutamate also plays an important role in the synthesis of functional folate (vitamin B9), which is a cofactor for one-carbon metabolism. Folate is predominantly decorated with a polyglutamate tail. The addition of polyglutamate to folate may enhance its co-enzyme affinity, subcellular compartmentation and stability [12].

In humans, glutamate and its metabolite GABA are important neurotransmitters in the central nervous system. Glutamate mainly employs its action through glutamate receptors [13], which also exist in non-neuronal tissues [14–16]. Thus, the functions of glutamate signaling may go beyond the nervous system [14–16]. Interestingly, plants also have glutamate receptor (GLR) homologs [17]. There are 20 *GLR* genes grouped into three clades in the model plant *Arabidopsis thaliana* [18]. The functions of these GLRs have just begun to be elucidated. Accumulating evidences suggest that plant GLRs may not have ligand specificity [19]. For instance, AtGLR1.4 is an ion channel gated by multiple hydrophobic amino acids but not glutamate [20]. Thus, GLRs may have evolved to have diverse functions in plants. Nevertheless, the discovery of GLR homologs has laid the foundation for the assessment of glutamate sensing and signaling in plants.

Glutamate has been implicated to modulate calcium signaling [21] and root system architecture [22, 23]. Glutamate inhibits primary root growth and stimulates the outgrowth of lateral roots near the primary root tip in Arabidopsis [22]. This phenomenon is specific to glutamate, as structurally or metabolically related amino acids Asp, Gln, and D-Glu do not have similar effects [22]. A recent study further demonstrated that a MAP kinase kinase kinase (MEKK1) is involved in glutamate signaling pathway responsible for inducing changes in Arabidopsis root system architecture [24]. The MAP kinase cascade plays an important role in both biotic and abiotic stress signaling networks [25]. The identification of MEKK1 in glutamate signaling raises an interesting question whether amino acid signaling interacts with biotic and abiotic stress signaling in plants. Recently, exogenous glutamate (10 mM) has been shown to induce systemic disease resistance in rice but the underlying molecular mechanisms are still unknown [26].

While glutamate has been shown to serve as an external signal to affect root growth and development in the most sensitive Arabidopsis accession C24 at a very low concentration (50 μM) [22], most studies on the effects of glutamate on the growth of seedlings or suspension cultures use 1–10 mM or even higher concentrations of glutamate [19]. It has been demonstrated that feeding of 20 or 40 mM glutamate to tobacco plants has limited effect on the endogenous glutamate pool [27, 28]. Feeding of 100 mM glutamate induces the expression of glutamate metabolic genes *cytosolic glutamine synthetase* (*GS1*) and *glutamate dehydrogenase* (*GDH*) in tobacco leaf discs [29]. Together with studies on glutamate metabolism related enzymes using inhibitors, mutants, overexpression and antisense lines, it has been proposed that plants may have mechanisms to maintain glutamate homeostasis [5]. GS and GDH may be responsible for maintaining a constant concentration of glutamate in plants [30].

Amino acids have been shown to act as signals to regulate gene expression in yeast and animals [31, 32]. It is somewhat surprising that relatively few studies have focused on the effects of exogenous amino acids on plant gene expression [19]. We have previously shown that glutamine can effectively support rice seedling growth when supplemented as the sole nitrogen source in hydroponics [33]. In addition to its role in plant nutrition, glutamine can rapidly induce the expression of key transcription factor genes involved in nitrogen and stress responses in rice roots [33, 34]. These findings support the notion that amino acid signaling pathways may crosstalk with biotic and abiotic signaling networks in plants. Although glutamate and glutamine are closely related in structure and metabolism, these two amino acids may have distinct signaling effects. Here, we examined the nutritional effects of glutamate on rice seedlings. We also used transcriptome analysis to identify genes that were rapidly induced by glutamate in rice roots. Some of the early glutamate-responsive genes identified here may be involved in glutamate signaling in plants.

## Results

### Exogenous glutamate can support rice seedling growth

**Fig. 1:**
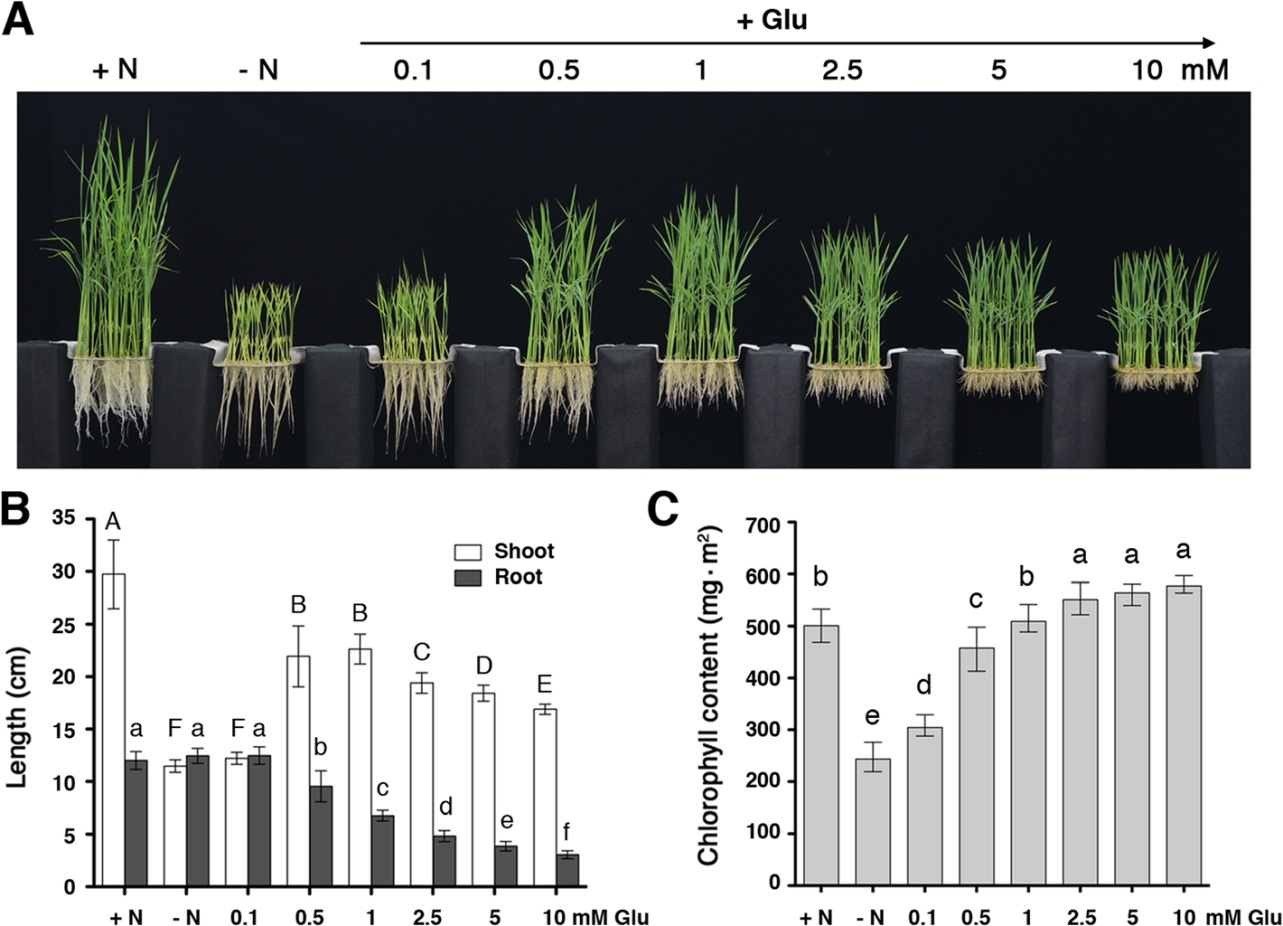
Glutamate as a nitrogen nutrient to support rice seedling growth. **a** Rice seedlings grown in hydroponics containing NH_4_NO_3_ or glutamate as the nitrogen source. Shoot length, root length (**b**), and chlorophyll contents (**c**) of rice seedlings from (**a**). The rice seedlings are 17-day-old. Data are means ± SD (*n* = 40). Different letters indicate significant differences between treatments, tested by one-way ANOVA followed by Tukey’s test (*P* < 0.05). +N, + 1.43 mM NH_4_NO_3_; −N, no nitrogen

Glutamate occupies a central position in plant metabolism and serves as a precursor for many important compounds (Additional file 1: Figure S1). To examine the nutritional effect of glutamate, we grew rice seedlings in hydroponics supplemented with different concentrations of glutamate as the sole nitrogen source (Fig. 1). Feeding of 0.1 mM glutamate did not significantly (one-way ANOVA followed by Tukey’s test, *P* < 0.05) improve the growth of rice seedlings (Fig. 1a,b), and the chlorophyll content only increased slightly (Fig. 1c) as compared with those of seedlings grown in the absence of nitrogen. The shoot length of rice seedlings grown in 0.5 and 1 mM glutamate was significantly longer than that grown in the absence of nitrogen, but was still shorter than that grown in 1.43 mM NH_4_NO_3_ (Fig. 1a,b). Supplementation of 2.5, 5, or 10 mM glutamate in the hydroponics inhibited shoot growth as compared with that of 0.5 or 1 mM glutamate (Fig. 1a,b). The root length of rice seedlings grown in 0.1 mM glutamate was similar to that of seedlings grown in the absence of nitrogen (Fig. 1a,b). Feeding of 0.5, 1, 2.5, 5, or 10 mM glutamate in the hydroponics increasingly inhibited root growth as compared with that of 1.43 mM NH_4_NO_3_ (Fig. 1a,b). Although glutamate was not as effective as ammonium nitrate in supporting rice seedling growth, the chlorophyll content in seedlings grown in 0.5-10 mM glutamate was comparable to that of seedlings grown in 1.43 mM NH_4_NO_3_ (Fig. 1c).

### Slow uptake of glutamate in nitrogen-starved rice seedlings

**Fig. 2:**
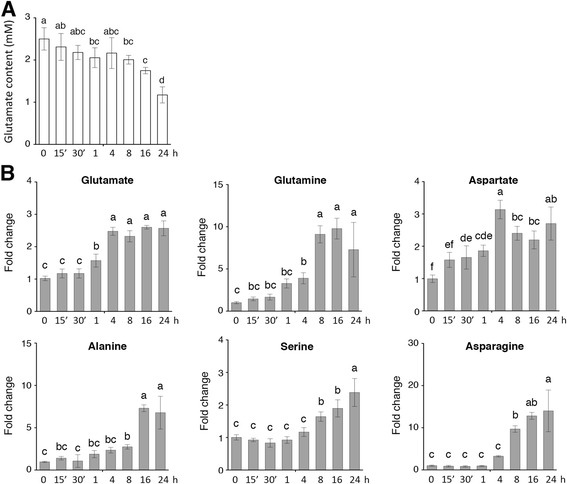
Amino acid contents in the growth medium and rice roots during the time course of glutamate treatment. 17-day-old nitrogen-starved rice seedlings were transferred to hydroponics containing 2.5 mM glutamate for 24 h. **a** The contents of glutamate left in the hydroponics were measured over the 24-h time course. **b** Contents of glutamate, glutamine, aspartate, alanine, serine, and asparagine in the roots were measured during the time course of glutamate treatment. Fold change indicates the relative amounts of amino acids in 2.5 mM glutamate-treated roots (0.25-24 h) compared to those of control (0 h). Data are means ± SD (*n* = 5). Different letters indicate significant differences between treatments, tested by one-way ANOVA followed by Tukey’s test (*P* < 0.05)

To examine if rice seedlings could effectively take up glutamate, 17-day-old nitrogen-starved rice seedlings were transferred to hydroponics containing 2.5 mM glutamate for 0–24 h. The content of glutamate left in the growth medium was measured during the time course of glutamate feeding. The amount of glutamate left in the growth medium only decreased slightly after 0.25-8 h of treatment (Fig. 2a). Approximately 20% and 50% of the glutamate supplemented in the hydroponics were consumed after 16 h and 24 h of treatment, respectively (Fig. 2a).

### Glutamate is rapidly converted to other amino acids in the roots

After taken up by the nitrogen-starved rice seedlings, glutamate may be converted to other nitrogen-containing compounds. We analyzed the levels of free amino acids in the roots during the time course of glutamate treatment. The levels of glutamate, aspartate, serine, glutamine, asparagine, and alanine increased significantly (one-way ANOVA followed by Tukey’s test, *P* < 0.05) after 24 h of glutamate treatment as compared to the untreated control (Fig. 2b, Additional file 1: Table S1). By contrast, the amounts of the other proteinogenic amino acids did not change significantly (data not shown). Interestingly, feeding of glutamate to nitrogen-starved rice seedlings did not significantly increase the endogenous levels of glutamate within 30 min (Fig. 2b). Glutamate in the roots started to accumulate to a higher level after feeding for 1 h and increased to about 2.5-fold of control levels after 4 h of glutamate treatment (Fig. 2b). Although the exogenous amount of glutamate in the growth medium decreased significantly after 8–24 h (Fig. 2a), the endogenous levels of glutamate in the roots did not further increase after 8–24 h of glutamate treatment (Fig. 2b). These results suggest that the glutamate taken up by the rice seedlings may be constantly converted to the other nitrogen-containing compounds in the roots.

The amount of glutamine, aspartate and alanine increased rapidly after 15 min of glutamate treatment (Fig. 2b). Feeding of glutamate to nitrogen-starved rice seedlings significantly increased the endogenous levels of glutamine after 15–30 min (Fig. 2b). The amount of glutamine in the roots increased to about 3-fold after 1 h, ~5-fold after 4 h, and continued to increase to ~10-fold of control levels after 8–24 h of glutamate treatment (Fig. 2b). Similar trend was observed in changes of alanine levels during the time course of glutamate treatment. The amount of alanine increased to about 2.5- to 3-fold after 4–8 h, and continued to increase to ~8-fold of control levels after 16–24 h of glutamate treatment (Fig. 2b). Feeding of glutamate to nitrogen-starved rice seedlings rapidly enhanced the accumulation of aspartate within the first hour, and the levels of aspartate increased to about 2- to 3.3-fold of control levels after 4–24 h of glutamate treatment (Fig. 2b).

By contrast, feeding of glutamate to nitrogen-starved rice seedlings did not significantly increase the amount of serine in the roots within the first 4 h (Fig. 2b). The levels of serine increased to about 2- to 3-fold of control levels after 8–24 h of glutamate treatment (Fig. 2b). The amount of asparagine was low in nitrogen-starved seedlings, and feeding of glutamate for 0.25-4 h did not significantly increase the levels of asparagine in the roots (Fig. 2b). The amount of asparagine started to increase significantly (~12-fold) after 8 h of glutamate treatment, and continued to increase to ~16-fold after 16 h, and ~20-fold of control levels after 24 h (Fig. 2b).

### Identification of early glutamate-responsive genes in rice seedlings

In addition to nutritional effects, we attempted to explore the signaling role of glutamate in the regulation of gene expression in rice. To identify genes that were rapidly induced by glutamate, we used microarray analysis to compare the gene expression profiles between rice seedlings treated with 2.5 mM glutamate for 30 min and the untreated control. Analysis of the microarray data with 2-fold cutoff revealed that the expression of 156 genes was rapidly regulated by glutamate in the roots. Of the 156 genes, including 151 up- and 5 down-regulated, we were able to confirm the up-regulation of 122 genes (Table 1), and none of the down-regulated genes could be verified by qRT-PCR (see below). In contrast to roots, the microarray data did not identify any genes that were rapidly induced by glutamate in the shoots (2-fold cutoff, data not shown). This is consistent with the finding that levels of free amino acids did not change significantly (Student’s *t*-test, *P* < 0.05) in the shoots after 30 min of glutamate treatment (Additional file 1: Figure S2A). Unexpectedly, the treatment also did not significantly increase the levels of glutamate in the roots, while the amounts of glutamine, aspartate, and alanine increased slightly (Additional file 1: Figure S2B).

**Table 1:** List of early glutamate-responsive genes in rice roots

No.	Locus ID		Fold change (+ Glu/- N)	Gene description
1^a^	Os03g0236200	LOC_Os03g13300	9.6	Glutamate decarboxylase 1, GDC1
2	Os08g0508800	LOC_Os08g39840	5.4	Herbivore induced 13-lipoxygenase, HI-LOX
3	Os12g0518200	LOC_Os12g33300	5.1	EamA-like transporter family
4	Os04g0618400	LOC_Os04g52750	5.1	Unknown
5^a^	Os03g0823400	LOC_Os03g60840	4.7	Bowman-Birk type trypsin inhibitor, BBTI13
6	Os09g0401000	LOC_Os09g23620	4.6	MYB family transcription factor
7^a^	Os05g0402900	LOC_Os05g33400	4.3	Xylanase inhibitor I-like
8	Os01g0186900	LOC_Os01g09220	4.2	Putative nuclease HARBI1
9	Os08g0457200	LOC_Os08g35580	4.2	Unknown
10	Os05g0373300	LOC_Os05g30970	3.9	Copine-like protein; similar to BONZAI1
11	Os01g0952900	LOC_Os01g72360	3.8	Unknown
12	Os01g0705200	LOC_Os01g50910	3.7	Late embryogenesis abundant protein, group 3
13	Os12g0181500	LOC_Os12g08090	3.7	Amino acid permease 16, AAP16
14	Os01g0666000	LOC_Os01g47580	3.6	Lipid phosphate phosphatase 2
15	Os03g0318400	LOC_Os03g20290	3.6	Aspartic proteinase nepenthesin-1
16^a^	Os04g0301500	LOC_Os04g23550	3.6	Transcription factor bHLH35, RERJ1
17	Os02g0269600	LOC_Os02g16940	3.6	Subtilisin-like protease SBT3.5
18	Os09g0565300	LOC_Os09g39190	3.5	E3 ubiquitin-protein ligase RGLG1
19	Os10g0542900	LOC_Os10g39680	3.5	Chitinase 8
20	Os02g0605900	LOC_Os02g39330	3.4	Chitinase 6
21	Os09g0551000	LOC_Os09g37834	3.3	G-type lectin S-receptor-like protein kinase RKS1
22^a^	Os01g0845100	LOC_Os01g62670	3.3	Protein of unknown function DUF668
23	Os11g0213800	LOC_Os11g10770	3.2	NBS-LRR disease resistance protein
24	Os03g0302800	LOC_Os03g19070	3.2	Unknown
25^a^	Os02g0687200	LOC_Os02g46210	3.1	Protein of unknown function DUF581
26	Os01g0905300	LOC_Os01g67820	3.1	Exo70 exocyst complex subunit
27	Os10g0497700	LOC_Os10g35460	3.1	COBRA-like protein 4
28	Os02g0733900	LOC_Os02g50110	3.1	Unknown
29	Os04g0585000	LOC_Os04g49550	3.0	RING-H2 finger protein ATL44
30	Os05g0516700	LOC_Os05g44060	3.0	Unknown
31^a^	Os03g0187800	LOC_Os03g08880	3.0	Purine permease 3, PUP3
32	Os04g0647900	LOC_Os04g55420	3.0	LRR receptor-like serine/threonine protein kinase GSO1
33	Os01g0705700		2.9	Transcription factor bHLH13
34	Os10g0418100	LOC_Os10g28240	2.9	Calcium-transporting ATPase 13
35	Os07g0559700	LOC_Os07g37320	2.9	Monosaccharide transporter 6, OsMST6
36	Os11g0144900	LOC_Os11g04830	2.9	Unknown
37	Os04g0464100	LOC_Os04g39010	2.8	Heavy metal transport domain-containing protein
38	Os09g0471800	LOC_Os09g29600	2.8	Wall-associated receptor kinase 85, OsWAK85
39	Os12g0478400	LOC_Os12g29430	2.8	Wall-associated receptor kinase 125, OsWAK125
40	Os04g0128700	LOC_Os04g03920	2.8	Unknown
41	Os07g0592600	LOC_Os07g40290	2.8	Indole-3-acetic acid-amido synthetase 3.8, OsGH3.8
42	Os01g0720400	LOC_Os01g52230	2.7	Inorganic pyrophosphatase 1
43	Os02g0764700		2.7	Ethylene-responsive transcription factor ERF109
44	Os01g0915000	LOC_Os01g68650	2.7	Protein of unknown function DUF506
45	Os01g0121500	LOC_Os01g03130	2.7	Unknown
46^a^	Os02g0807900	LOC_Os02g56380	2.7	Wall-associated receptor kinase 21, OsWAK21
47	Os04g0543900	LOC_Os04g45970	2.7	Glutamate dehydrogenase 2, GDH2
48	Os03g0203700	LOC_Os03g10640	2.7	Calcium-transporting ATPase 2
49	Os12g0198200	LOC_Os12g09640	2.7	Phosphatase 2C family protein
50	Os04g0463500	LOC_Os04g38950	2.7	Anthranilate synthase beta subunit 1
51	Os05g0540900	LOC_Os05g46340	2.7	Unknown
52	Os08g0473900	LOC_Os08g36910	2.7	Alpha-amylase isozyme 3D
53	Os01g0717000	LOC_Os01g51920	2.7	Choline kinase 2
54	Os07g0493800	LOC_Os07g31190	2.7	Wall-associated receptor kinase 71, OsWAK71
55	Os05g0181300	LOC_Os05g08860	2.6	Unknown
56	Os11g0667700	LOC_Os11g44560	2.6	Protein kinase domain containing protein
57^a^	Os02g0205500	LOC_Os02g11070	2.6	3-ketoacyl-CoA synthase 11
58	Os03g0290300	LOC_Os03g18070	2.6	Omega-3 fatty acid desaturase
59	Os06g0201200	LOC_Os06g10020	2.6	Unknown
60	Os01g0905200	LOC_Os01g67810	2.6	Exo70 exocyst complex subunit
61	Os03g0268600	LOC_Os03g16170	2.6	Phosphatase 2C family protein
62	Os04g0618700	LOC_Os04g52780	2.6	LRR receptor-like serine/threonine protein kinase FLS2
63	Os12g0556200	LOC_Os12g36910	2.6	Calmodulin binding protein 60 B
64	Os11g0474533	LOC_Os11g28470	2.5	Unknown
65	Os04g0119500	LOC_Os04g02910	2.5	Unknown
66	Os12g0248600	LOC_Os12g14540	2.5	Unknown
67^a^	Os04g0194500	LOC_Os04g11820	2.5	ABC transporter G family member 28
68	Os04g0497000	LOC_Os04g41960	2.5	NADPH oxidoreductase
69	Os03g0648600	LOC_Os03g44636	2.5	RING-H2 finger protein ATL44-like
70	Os11g0154500	LOC_Os11g05614	2.5	NAC-domain containing protein 90
71^a^	Os02g0585100	LOC_Os02g37320	2.5	Heavy metal transport domain containing protein
72	Os09g0313600	LOC_Os09g14450	2.4	Disease resistance RPP13-like protein 4
73	Os08g0457000	LOC_Os08g35560	2.4	Unknown
74	Os01g0776700	LOC_Os01g56890	2.4	Unknown
75	Os11g0168600	LOC_Os11g06780	2.4	Leucine-rich repeat receptor protein kinase MSP1-like
76	Os09g0455300	LOC_Os09g28210	2.4	bHLH transcription factor, similar to HECATE1 (HEC1)
77	Os09g0484900	LOC_Os09g31130	2.3	Tonoplast dicarboxylate transporter
78	Os09g0452900	LOC_Os09g27950	2.3	Beta-1,3-galactosyltransferase 7
79	Os03g0292100	LOC_Os03g18150	2.3	Phosphatase 2C family protein
80	Os05g0493100	LOC_Os05g41370	2.3	Cysteine-rich receptor-like protein kinase 15
81	Os11g0228600	LOC_Os11g12240	2.2	Similar to NBS-LRR disease resistance protein
82	Os04g0490500	LOC_Os04g41310	2.2	PTI1-like tyrosine-protein kinase 3
83	Os03g0194600	LOC_Os03g09880	2.2	Cyt b561 and DOMON domain-containing protein
84	Os01g0134700	LOC_Os01g04280	2.2	Calmodulin binding protein
85	Os02g0661100	LOC_Os02g44230	2.2	Trehalose-6-phosphate phosphatase
86	Os10g0521900	LOC_Os10g37760	2.2	Rhomboid-like protease, OsRhmbd17
87	Os08g0384500	LOC_Os08g29570	2.2	ABC transporter G family member 44
88	Os03g0218400	LOC_Os03g11900	2.2	Sugar transport protein 2
89	Os04g0461600	LOC_Os04g38790	2.2	Cell number regulator 2
90	Os02g0627100	LOC_Os02g41680	2.2	Phenylalanine ammonia-lyase
91	Os03g0407900	LOC_Os03g29410	2.2	Serine/threonine protein kinase
92	Os07g0502200	LOC_Os07g31884	2.2	MATE efflux protein family protein
93	Os02g0562600	LOC_Os02g35490	2.2	MLO-like protein 1
94	Os04g0634700	LOC_Os04g54200	2.1	Diacylglycerol kinase 5
95	Os01g0882800	LOC_Os01g66010	2.1	Amino acid permease 8, AAP8
96	Os07g0232800	LOC_Os07g12890	2.1	Zinc transporter 8
97^a^	Os09g0482800	LOC_Os09g30490	2.1	Calcium-binding EF-hand domain containing protein
98	Os03g0773300	LOC_Os03g56250	2.1	LRR receptor-like serine/threonine protein kinase
99	Os01g0934400	LOC_Os01g70820	2.1	Photosystem II oxygen evolving complex protein PsbP
100^a^	Os08g0138200	LOC_Os08g04370	2.1	Cupredoxin domain containing protein, phytocyanin
101	Os01g0690800	LOC_Os01g49614	2.1	Acidic endochitinase SE2
102	Os03g0792800	LOC_Os03g57880	2.1	Glucan endo-1,3-beta-glucosidase 8
103	Os05g0541100	LOC_Os05g46350	2.1	IQ calmodulin-binding region domain containing protein
104	Os01g0817000	LOC_Os01g60110	2.1	Protein of unknown function DUF607
105	Os01g0723800	LOC_Os01g52550	2.1	ABC transporter B family member 8
106	Os07g0561800	LOC_Os07g37454	2.1	Organic cation/carnitine transporter 2
107	Os01g0713200	LOC_Os01g51570	2.1	Glucan endo-1,3-beta-glucosidase GII
108	Os02g0126400	LOC_Os02g03410	2.1	Calcium-dependent protein kinase 16
109^a^	Os07g0119300	LOC_Os07g02800	2.1	MYB domain containing protein
110	Os02g0682300	LOC_Os02g45780	2.1	E3 ubiquitin-protein ligase RHA1B
111	Os07g0583600	LOC_Os07g39470	2.1	Chitin-inducible gibberellin-responsive protein 2, CIGR2
112^a^	Os11g0184900	LOC_Os11g08210	2.0	NAC domain-containing protein 5, OsNAC5
113	Os01g0570800	LOC_Os01g38980	2.0	IQ calmodulin-binding region domain containing protein
114	Os10g0466800	LOC_Os10g32930	2.0	Unknown
115	Os04g0632100	LOC_Os04g53998	2.0	Receptor-like serine/threonine-protein kinase SD1-6
116	Os06g0288100	LOC_Os06g18000	2.0	Leucine-rich repeat receptor-like protein kinase SOBIR1
117	Os02g0299300	LOC_Os02g19650	2.0	Putative aminoacrylate hydrolase RutD
118^a^	Os07g0589000	LOC_Os07g40000	2.0	LOB domain containing protein, LBD37-like
119	Os03g0285800	LOC_Os03g17700	2.0	MAP Kinase 5
120	Os06g0292400	LOC_Os06g18900	2.0	Unknown
121	Os08g0492500	LOC_Os08g38460	2.0	Probable E3 ubiquitin-protein ligase XERICO
122^a^	Os08g0386200	LOC_Os08g29660	2.0	WRKY69

Total RNA extracted from 17-day-old rice seedlings grown in hydroponic solution without nitrogen (−N) or treated with 2.5 mM glutamate for 30 min (+ Glu) was used for microarray analysis. ^a^Indicates genes that are also rapidly induced by glutamine [33]. The results were derived from two biological replicates

**Fig. 3:**
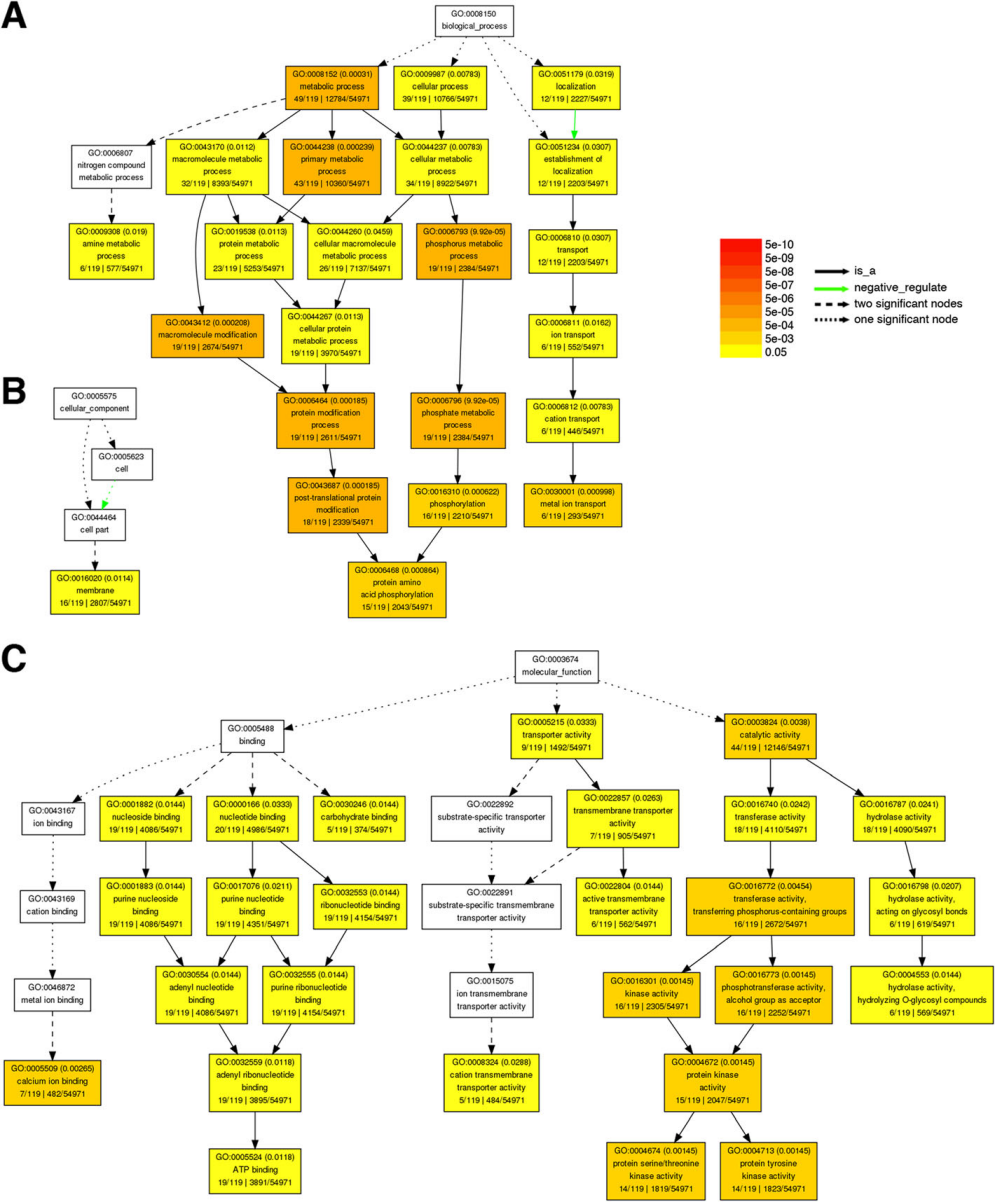
Gene ontology enrichment analysis of genes up-regulated by glutamate. The differentially expressed genes were analyzed by singular enrichment analysis using AgriGO. Significantly enriched GO categories in biological process (**a**), cellular component (**b**), and molecular function (**c**) are shown in yellow, orange, and red boxes (false discovery rate, FDR < 0.05). Each box contains GO term number, the FDR value, GO term, and number associated with the GO term in the query list and glutamate up-regulated genes (119 GO terms) as well as total number of query list and reference background (54971 GO terms). The GO terms “primary metabolic process”, “transporter activity” and “protein kinase activity” are significantly enriched in glutamate-responsive genes

We used gene ontology (GO) category enrichment analysis to classify the biological functions of 122 genes up-regulated by glutamate in rice roots. In biological process, the GO terms “metal ion transport”, “protein amino acid phosphorylation”, and “amine metabolic process” were significantly (false discovery rate value, FDR < 0.05) enriched (Fig. 3a). In cellular component, the GO term “membrane” was enriched (Fig. 3b). In molecular function, the GO terms “hydrolase activity”, “protein kinase activity”, “active transmembrane transporter activity”, “cation transmembrane transporter activity”, “ATP binding” and “calcium ion binding” were significantly enriched (Fig. 3c). Some of the glutamate up-regulated genes in representative functional categories were listed in Additional file 1: Table S2.

In addition, we also performed Kyoto encyclopedia of genes and genomes (KEGG) analysis. Of the 122 up-regulated genes, 33 genes were annotated with KEGG orthology (KO) terms. A list of these genes, the associated KO number and KEGG pathways were shown in Additional file 1: Table S3. We further performed KEGG pathway enrichment analysis and the result indicated that “glycerophospholipid metabolism” and “ABC transporters” were enriched (Additional file 1: Table S4). Since the gene count was very low, 2 in “glycerophospholipid metabolism” and only 1 in “ABC transporters”, the result of KEGG pathway enrichment analysis might not be meaningful. Nevertheless, the results of GO and KEGG analyses suggest that glutamate feeding for 30 min can rapidly trigger the expression of genes involved in metabolism, transport and signaling in rice roots.

The functions of the early glutamate-responsive genes are very diverse. Of the 122 genes identified, at least 11 genes encode putative transcription factors. The *Os07g0589000* gene encodes a homolog of Arabidopsis LBD37 that is involved in the regulation of nitrogen response [35]. *CIGR2* (*Os07g0583600*), an elicitor-responsive gene, encodes a GRAS family protein that has been shown to suppress cell death in rice [36]. NAC5 (Os11g0184900), no apical meristem protein 5, is involved in abiotic stress responses [37–39]. The expression of *Os04g0301500* (*basic helix-loop-helix 35, bHLH35*) is rapidly induced by jasmonate, and thus has been named *RERJ1* [40–42]. The other glutamate-responsive transcription factor genes include *Os09g0401000* (*MYB family protein*), *Os01g0705700* (*bHLH13*), *Os02g0764700* (*ERF109*), *Os11g0154500* (*NAC90*), *Os09g0455300* (*bHLH*, similar to *HECATE1*), *Os07g0119300* (*MYB family protein*), and *Os08g0386200* (*WRKY69*).

The expression of *Os03g0236200* (*glutamate decarboxylase 1*, *GDC1*) and *Os04g0543900* (*glutamate dehydrogenase 2*, *GDH2*) was rapidly induced by glutamate (Table 1). The enzymes encoded by these two genes are directly involved in glutamate metabolism. In addition to genes related to metabolism and transport, many genes involved in signal transduction, growth regulation, defense and stress responses were also rapidly induced by glutamate (Table 1). For instance, the expression of several genes encoding kinases, phosphatases, and calcium signaling related proteins was rapidly induced by glutamate (Table 1). The cell wall associated kinases (WAKs) may serve as pectin receptors to regulate plant growth and stress responses [43, 44]. Interestingly, glutamate rapidly induced the expression of several *WAK* genes (Table 1). The indole-3-acetic acid-amido synthetase OsGH3.8 functioning in auxin-dependent development can promote salicylate- and jasmonate-independent basal immunity in rice [45]. The expression of *OsGH3.8* (*Os07g0592600*) was rapidly and strongly induced by different concentrations of glutamate (Table 1, Additional file 1: Figure S3, no. 41). Several defense-related genes such as *herbivore induced 13-lipoxygenase* (*HI-LOX*, *Os08g0508800*), *chitinase 6* (*Os02g0605900*) and *8* (*Os10g0542900*) were also rapidly induced by glutamate (Table 1).

### Regulation of early glutamate-responsive genes by different concentrations of glutamate

**Fig. 4:**
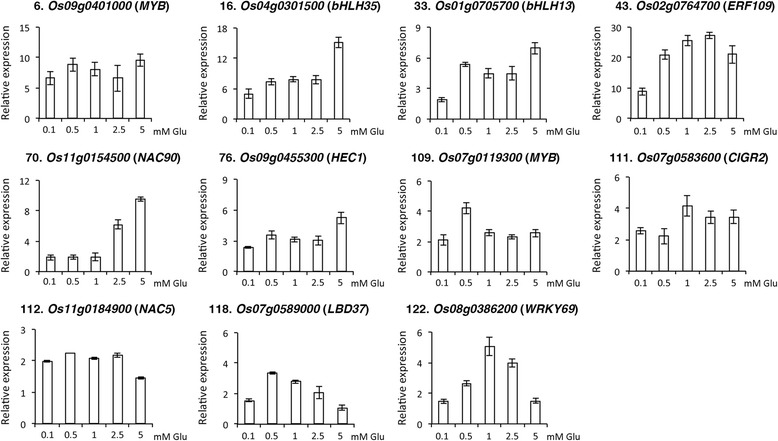
Regulation of glutamate-responsive transcription factor genes by different concentrations of glutamate. 17-day-old nitrogen-starved rice seedlings were transferred to hydroponics containing 0, 0.1, 0.5, 1, 2.5 and 5 mM glutamate for 30 min. Total RNA extracted from roots was used for qRT-PCR to analyze the expression of 11 glutamate-responsive transcription factor genes. The number of each gene corresponds to the number in Table 1. Relative expression indicates the fold-change of each gene as compared to that of control

To verify the microarray data, total RNA extracted from 17-day-old rice seedlings treated with 0–5 mM glutamate for 30 min was used for qRT-PCR analysis. We were able to confirm that glutamate (2.5 mM, 30 min) could induce the expression of 122 genes for more than 2-fold as compared to the untreated control. The effects of different concentrations of glutamate on the expression of these genes are shown in Fig. 4 and Additional file 1: Figure S3. In addition to verifying the microarray data, the results could also reveal the sensitivity and dosage dependence of these genes to glutamate. For instance, the expression of *Os09g0401000* (*MYB family protein*), *Os04g0301500* (*bHLH35*) and *Os02g0764700* (*ERF109*) was very sensitive to glutamate as treatment of 0.1 mM glutamate for 30 min resulted in greater than 5-fold induction in these genes as compared to the untreated control (Fig. 4). The other genes that are sensitive to glutamate induction include *Os03g0236200* (*GDC1*), *Os01g0705200* (*late embryogenesis abundant protein*), *Os12g0181500* (*amino acid permease 3*), *Os02g0687200* (*unknown*), *Os07g0592600* (*OsGH3.8*), *Os01g0720400* (*inorganic pyrophosphatase 1*), *Os11g0474533* (*unknown*), *Os02g0627100* (*phenylalanine ammonia-lyase*), and *Os09g0482800* (*EF-hand domain containing protein*). The expression of these genes was strongly induced by 0.1 mM glutamate and stayed at high levels (>5-fold) or continued to increase when treated with higher concentrations of glutamate as compared to the untreated control (Additional file 1: Figure S3).

### Regulation of glutamate-responsive transcription factor genes by different nitrogen

**Fig. 5:**
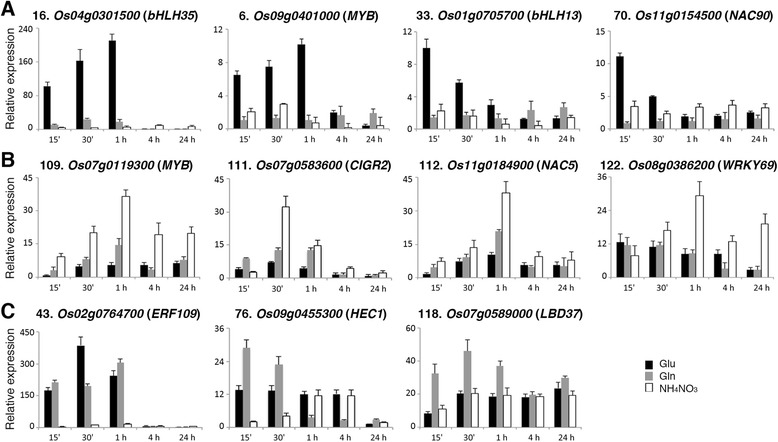
Effects of glutamine and NH_4_NO_3_ on the expression of glutamate-responsive transcription factor genes *bHLH35*, *MYB* (*Os09g0401000*), *bHLH13*, *NAC90* (**a**), *MYB* (*Os07g0119300*), *CIGR2*, *NAC5*, *WRKY69* (**b**), *ERF109*, ﻿*HEC1* and *LBD37* (**c**). 17-day-old nitrogen-starved rice seedlings were transferred to hydroponics containing 2.5 mM glutamate, glutamine, or 1.43 mM NH_4_NO_3_ for 0, 15 min, 30 min, 1, 4, and 24 h. Total RNA extracted from roots was used for qRT-PCR to analyze the expression of 11 glutamate-responsive transcription factor genes. The number of each gene corresponds to the number in Table 1. Relative expression indicates the fold-change of each gene as compared to that of control

To further examine if the response was specific to glutamate, we compared the effects of glutamate, glutamine, and ammonium nitrate on the expression of 11 glutamate-responsive transcription factor genes. Total RNA extracted from nitrogen-starved rice seedlings treated with 2.5 mM glutamate, glutamine, or 1.43 mM ammonium nitrate for 15 min to 24 h was used for qRT-PCR analysis. The results revealed that glutamine and ammonium nitrate could rapidly induce the expression of many glutamate-responsive transcription factor genes in the roots (Fig. 5). Among these transcription factor genes, the expression of *bHLH35* (*Os04g0301500*) was rapidly and strongly induced by glutamate (~90-fold, 15 min; ~160-fold, 30 min; ~230-fold, 1 h) but was not or only slightly induced by glutamine and ammonium nitrate (Fig. 5a). The expression of *MYB* (*Os09g0401000*), *bHLH13* (*Os01g0705700*) and *NAC90* (*Os11g0154500*) was preferentially induced by glutamate within 15–30 min of treatments (Fig. 5a). By contrast, the expression of another *MYB* (*Os07g0119300*) was rapidly and preferentially induced by ammonium nitrate (Fig. 5b). The expression of *CIGR2* (*Os07g0583600*) was strongly induced by ammonium nitrate after treatment for 30 min (Fig. 5b). Although the expression of *NAC5* (*Os11g0184900*) and *WRKY69* (*Os08g0386200*) was induced by all nitrogen treatments, ammonium nitrate seemed to have stronger effects on the induction of these genes (Fig. 5b). While ammonium nitrate had little effect on the induction of *ERF109* (*Os02g0764700*), glutamate and glutamine rapidly and strongly induced the expression of *ERF109* (Fig. 5c). The expression of *HEC1* (*Os09g0455300*) and *LBD37* (*Os07g0589000*) was preferentially induced by glutamine (Fig. 5c).

### Identification of genes that are specifically induced by glutamate

**Fig. 6:**
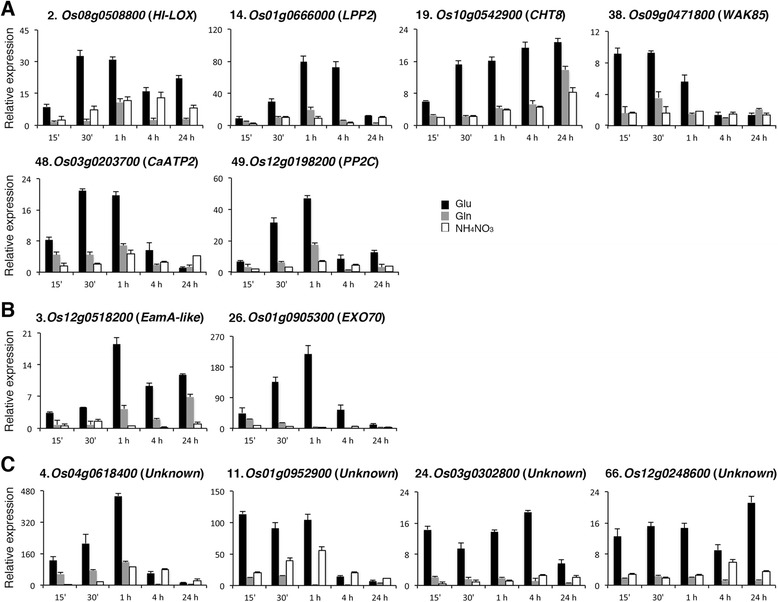
qRT-PCR analyses of genes that are specifically or preferentially induced by glutamate. 17-day-old rice seedlings grown in hydroponics without nitrogen were subsequently transferred to medium containing 2.5 mM glutamate, glutamine, or 1.43 mM NH_4_NO_3_ for 0, 15 min, 30 min, 1, 4, and 24 h. Total RNA extracted from roots was used for qRT-PCR to analyze the expression of *Os08g0508800* (*herbivore induced 13-lipoxygenase, HI-LOX*), *Os01g0666000* (*lipid phosphate phosphatase 2, LPP2*), *Os10g0542900* (*chitinase 8, CHT8*), *Os09g0471800* (*wall associated kinase 85, WAK85*), *Os03g0203700* (*calcium transporting ATPase 2, CaATP2*), *Os12g0198200* (*phosphatase 2C, PP2C*) (**a**), *Os12g0518200* (*EamA-like transporter*), *Os01g0905300* (*exocyst 70 subunit, EXO70*) (**b**), and four unknown function genes *Os04g0618400*, *Os01g0952900*, *Os03g0302800* and *Os12g0248600* (**c**). The number of each gene corresponds to the number in Table 1. Relative expression indicates the fold-change of each gene as compared to that of control

The discovery that the expression of *bHLH35* (*Os04g0301500*), *MYB* (*Os09g0401000*), *bHLH13* (*Os01g0705700*) and *NAC90* (*Os11g0154500*) was specifically or preferentially induced by glutamate prompted us to examine the expression of the other 111 glutamate-responsive genes under glutamate, glutamine and ammonium nitrate time course treatments. The expression of many glutamate-responsive genes was also rapidly induced by glutamine and ammonium nitrate as compared with that of nitrogen-starved rice seedlings (Additional file 1: Figure S4). However, the expression patterns and the amounts of transcripts accumulated in response to different nitrogen sources varied from gene to gene. Of the additional 111 genes examined, the expression of at least 12 genes was strongly and preferentially induced by glutamate (Fig. 6). Interestingly, except the unknown function genes, most of the genes preferentially induced by glutamate, e.g. *Os08g0508800* (*HI-LOX*), *Os01g0666000* (*lipid phosphate phosphatase 2*), *Os10g0542900* (*chitinase 8*), *Os09g0471800* (*WAK 85*), *Os03g0203700* (*calcium transporting ATPase 2*), *Os12g0198200* (*phosphatase 2C*), are related to signal transduction or defense responses (Fig. 6a).

The expression of *Os12g0518200* (*EamA-like transporter*) and *Os01g0905300* (*exocyst 70 subunit*) was rapidly and preferentially induced by glutamate (Fig. 6b). The functions of these two genes are related to transport and secretion. The expression of at least 4 unknown function genes, e.g. *Os04g0618400*, *Os01g0952900*, *Os03g0302800* and *Os12g0248600*, was specifically or preferentially induced by glutamate (Fig. 6c).

### Glutamate rapidly induces the expression of *GDC1*

**Fig. 7:**
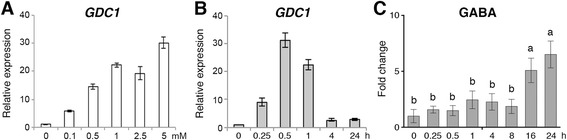
Effects of glutamate on the expression of *GDC1* and accumulation of GABA. qRT-PCR analysis of *GDC1* from roots of 17-day-old rice seedlings treated with different concentrations of glutamate for 30 min (**a**), or treated with 2.5 mM glutamate for 0.25-24 h (**b**). Relative expression indicates the fold-change of each gene as compared to that of control. **c** The amounts of GABA in the roots. 17-day-old nitrogen-starved rice seedlings were transferred to hydroponics containing 2.5 mM glutamate for 24 h. The amounts of GABA in the roots were measured during the time course of glutamate treatments. Fold change indicates the relative amount of GABA compared to that of control (0 h). Data are means ± SD (*n* = 4). Different letters indicate significant differences between treatments, tested by one-way ANOVA followed by Tukey’s test (*P* < 0.05)

It is interesting that the expression of *Os03g0236200* (*GDC1*) has the strongest induction (9.6-fold) by glutamate in the microarray analysis (Table 1). Treatment of different concentrations of glutamate (0.1-5 mM, 30 min) revealed that the expression of *GDC1* was very sensitive to glutamate as treatment of 0.1 mM glutamate already significantly induced the expression of *GDC1* (5.8-fold) as compared to the untreated control in the roots (Fig. 7a). Furthermore, the effect of glutamate on the induction of *GDC1* is dosage dependent, e.g. the induction is stronger as the concentration of glutamate increases (Fig. 7a). In addition, the results of glutamate time course treatments revealed that glutamate rapidly and strongly induced the expression of *GDC1* (Fig. 7b). The amount of *GDC1* transcripts increased approximately 20–30 folds after 0.5-1 h of 2.5 mM glutamate treatment as compared to the levels of the untreated control (Fig. 7b). The function of GDC is to convert glutamate to GABA. To further examine if the induction of *GDC1* might contribute to the accumulation of GABA, we measured the amount of GABA in the seedlings during the time course of glutamate treatment. In contrast to the rapid induction of the *GDC1* gene, the content of GABA did not increase significantly after 0.25 to 8 h of glutamate treatments as compared to the levels of the untreated control in the roots. The amount of GABA increased significantly (~5-7 folds) until 16 to 24 h of glutamate treatments (Fig. 7c).

## Discussion

### Nutritional effect of glutamate on rice

Although glutamate and glutamine are closely related, exogenous glutamine appears to be more effective than glutamate in supporting rice seedling growth. We previously showed that supplementation of 0.1 mM glutamine could significantly improve the growth of rice seedlings in hydroponics [33]. Here, we demonstrated that feeding of 0.1 mM glutamate had little effect and supplementation of 0.5 mM glutamate could significantly enhance rice seedling growth comparable to that of 0.1 mM glutamine (Fig. 1) [33]. The optimal concentration of exogenous glutamate to support rice seedling growth is around 0.5-1 mM. When the supplemented glutamate exceeds this amount, the excess glutamate will inhibit the growth of rice seedlings. Together, these results support the notion that glutamate can serve as a nitrogen nutrient, but it is not as effective as ammonium nitrate or glutamine.

As leaf nitrogen content and chlorophyll concentration are closely linked, the level of leaf chlorophyll is commonly used as an indicator of endogenous nitrogen status. The chlorophyll contents in rice seedlings grown in 2.5-10 mM glutamate were similar to those grown in ammonium nitrate. These results suggest that the rice seedlings grown in 2.5-10 mM glutamate can efficiently synthesize chlorophylls and do not have symptoms of nitrogen deficiency. Thus, the inhibitory effects of 2.5-10 mM glutamate on the growth of rice seedling are likely caused by over nutrition or glutamate toxicity, rather than nitrogen deficiency.

We previously showed that glutamine could be rapidly taken up by nitrogen-starved rice seedlings and was almost used up in hydroponics after 24 h of feeding [33]. Here, we performed a similar experiment and found that nitrogen-starved rice seedlings could not consume glutamate as effectively as glutamine. After feeding of glutamate to nitrogen-starved rice seedlings for 24 h, approximately 50% of the supplemented glutamate was still left in the growth medium (Fig. 2a). These results suggest that rice seedlings may have different mechanisms to absorb glutamine and glutamate. In Arabidopsis, four amino acid transporters, e.g. AAP1, AAP5, ProT2, and LHT1, have been shown to play a role in amino acid uptake by the root [46]. By contrast, amino acid transporters have been rarely studied in monocots [47]. Recently, analysis of rice amino acid permeases reveals that OsAAP1, OsAAP7 and OsAAP16 function as general amino acid permeases and transport all amino acids well except aspartate and β-alanine, whereas OsAAP3 has a distinct substrate specificity that prefers neutral and basic amino acids [48]. Interestingly, these rice AAPs all have better specificity to glutamine than glutamate [48]. It is likely that rice roots may have a more efficient transport system to take up glutamine than glutamate, which is consistent with our hydroponic feeding results.

### Glutamate homeostasis in rice seedlings

Glutamate is the most abundant free amino acid in nitrogen-starved rice seedlings (Additional file 1: Figure S2). Interestingly, feeding of 2.5 mM glutamate to nitrogen-starved rice seedlings did not significantly increase the amount of endogenous glutamate within the first hour. The glutamate content increased to approximately 2.5-fold of control after 4–24 h of feeding, which are relatively small as compared to those of glutamine (~10-fold) and GABA (~7-fold), two nitrogen-containing compounds directly linked to glutamate metabolism. Asparagine is a relative inert amino acid. Levels of asparagine increased to ~ 20-fold of control after 24 h of glutamate feeding. Asparagine and glutamine have high nitrogen to carbon ratios that play important roles in nitrogen storage and transport in plants. The accumulation of these amino acids indicates that the rice seedlings are not deficient of nitrogen after several hours of glutamate feeding.

We previously showed that feeding of glutamine to nitrogen-starved rice seedlings resulted in rapid and dramatic accumulation of glutamine, but not glutamate, in the roots [33]. Here, we demonstrated that feeding of glutamate also resulted in dramatic increases of glutamine, but not glutamate. These results suggest that glutamate, a very active amino acid, either directly absorbed from the environment or derived from glutamine, will be quickly metabolized to other nitrogen-containing compounds in plants. In addition to its critical role in metabolism, glutamate may also function as a signaling molecule to regulate plant growth and development. Thus, it is important for plants to maintain the homeostasis of glutamate as dramatic fluctuations of glutamate may have detrimental effects on plant metabolism, growth and development. It is not clear how plants maintain the homeostasis of glutamate. The rapid induction of glutamate metabolic genes such as *GDC1* (*Os03g0236200*) and *GDH2* (*Os04g0543900*) observed in this study may represent one of the strategies to maintain glutamate homeostasis. Still, other mechanisms involved in the regulation of glutamate homeostasis have yet to be uncovered in plants.

### Glutamate can trigger an elicitor-like response in plants

It is unexpected that many genes related to defense responses are rapidly induced by glutamate. For instance, the elicitor-responsive gene *CIGR2* encodes a transcriptional activator that is involved in hypersensitive response during pathogen infection [36]. The JA responsive gene *bHLH35* (*RERJ1*) is involved in disease resistance and drought tolerance [41, 49]. Herbivore-induced 13-lipoxygenase (OsHI-LOX) has been demonstrated to be involved in defense response [50]. The indole-3-acetic acid–amido synthetase GH3.8 is involved in salicylate- and jasmonate-independent basal immunity in rice [45]. Several wall-associated kinases are involved in basal defense against rice blast fungus [44]. Glutamate rapidly induced the expression of *CIGR2*, *OsHI-LOX*, *OsGH3.8*, *WAKs* and defense-related genes encoding trypsin inhibitor, xylanase inhibitor, aspartic proteinase, subtilisin-like protease, chitinase, and disease-related receptor-like protein kinases (Table 1). Glutamate also rapidly induced the expression of stress-related genes encoding late embryogenesis abundant (LEA) protein, E3 ubiquitin-protein ligase, heavy metal transport domain-containing protein, MATE efflux protein, phytocyanin, and glycosyl hydrolase (Table 1). The rapid induction of these defense- and stress-related genes suggests that glutamate may trigger an elicitor-like response in rice seedlings.

Interestingly, exogenous glutamate has been shown to induce systemic disease resistance in rice [26]. It is conceivable that glutamate may have a role similar to an elicitor or the exogenous glutamate may affect the cell wall and triggers an elicitor-like response in the plant cell. Glutamate or changes in the cell wall caused by exogenous glutamate may be perceived by receptor or sensor proteins located on the cell surface, which in turn transmit the signal to the nucleus to regulate the expression of defense-related genes. Alternatively, the endogenous glutamate or metabolites derived from glutamate may be directly involved in the regulation of defense-related genes.

In addition to defense and stress-related genes, glutamate also rapidly induced the expression of genes involved in metabolism, transport, growth and signal transduction.

Some of the early glutamate-responsive genes encode membrane/wall receptors, transporters, calcium signaling proteins, protein kinases/phosphatases, and transcription factors (Table 1, Additional file 1: Table S2), which may be involved in glutamate sensing and signaling in rice roots. Although the expression of glutamate receptor genes is not rapidly induced by glutamate (Additional file 1: Table S5), we cannot exclude the possibility that the glutamate signaling pathways are mediated by GLRs to regulate gene expression in rice roots. Still, glutamate may employ its signaling functions through GLR independent pathways. Some of the early glutamate-responsive genes identified in this study may be involved in the GLR dependent or independent pathways.

### Interactions between glutamate and glutamine signaling pathways

Glutamate and glutamine are closely related in structure and metabolism. Although glutamine is more effective in serving as a nitrogen nutrient, glutamate has more profound effects on the regulation of gene expression in rice seedlings. Glutamine rapidly induces the expression of ~35 genes [33], whereas glutamate induces the expression of at least 122 genes in rice roots. Some of the glutamate-induced genes are specifically related to glutamate metabolism and transport. For instance, the expression of *GDH2* and several transporter genes is induced by glutamate (Table 1). Glutamine induces the expression of *glutamine dumper* genes [33], which are not induced by glutamate. An unexpected common theme is that both glutamate and glutamine rapidly induce the expression of stress response genes. Glutamate, in particular, affects more genes related to defense function. Further studies on this newly emerging theme, e.g. amino acids and defense response, promise to provide new insights into the molecular mechanism of amino acid signaling in plants.

Still, the microarray data revealed that glutamate and glutamine commonly induced the expression of 17 genes (Table 1). Most of the commonly induced genes are not directly involved in metabolism. Interestingly, 5 of the 17 commonly induced genes encode putative transcription factors, e.g. bHLH35 (Os04g0301500), MYB (Os07g0119300), NAC5 (Os11g0184900), LBD37-like (Os07g0589000), and WRKY69 (Os08g0386200). It is possible that glutamate and glutamine may share some components in the signaling pathways to regulate plant growth and stress responses. Alternatively, some of the glutamate effects may be indirectly caused by glutamine as treatment of exogenous glutamate rapidly and significantly increases the amount of endogenous glutamine. Nevertheless, we have identified several genes that are specifically or preferentially induced by glutamate (Figs. 5, 6 and Additional file 1: Figure S4). These genes can be used to dissect the molecular mechanism of glutamate signaling and regulation of gene expression in the future.

### Significance of exogenous glutamate treatment

Nitrate and ammonium have been considered as the dominant nitrogen sources for plants and research on plant nitrogen nutrition has thus heavily focused on these inorganic nitrogen forms. One of the reasons that drives many researchers to study the effects of nitrate and ammonium on plants is the use of inorganic nitrogen fertilizers in agriculture. In fact, organic and inorganic nitrogen sources coexist in the ecosystem, and plants can use a diverse array of nitrogen forms, including amino acids, present in the soil [51]. It has been shown that Arabidopsis roots can take up amino acids at naturally occurring concentrations from agricultural soil [52, 53]. Under natural conditions, decomposing organic matters including plant and animal tissues may result in organic nitrogen-rich patches in the soil. Although glutamate concentrations are normally low (<10 μM) in bulk soil solutions [54], high concentrations of glutamate may routinely occur in organic nitrogen-rich patches as plant and animal tissues contain free glutamate at millimolar levels [55, 56]. The concentrations of apoplastic glutamate has been reported in the range of 0.3-1.3 mM in a variety of tissues and plant species [57–61]. Interestingly, some of the glutamate-responsive genes identified here can be rapidly induced (30 min) by exogenous glutamate at a relatively low concentration (0.1 mM). These results suggest that the signaling role of glutamate in the regulation of gene expression may occur *in planta*.

## Conclusion

Glutamate is a very active amino acid that occupies a central position in the primary metabolism in plants. Here, we show that glutamate, the most abundant amino acid in nitrogen-starved rice seedlings, may play a role in plant nutrition and function as a signaling molecule to regulate gene expression. In addition to genes involved in metabolism, transport, growth and signal transduction, glutamate rapidly induces the expression of genes related defense and stress responses. The elicitor-like response triggered by glutamate may partly explain the effect of exogenous glutamate on the induction of disease resistance in rice. The nutritional effects and the diverse functions of early glutamate-responsive genes support the notion that glutamate is an important metabolic fuel and a functional amino acid in plants.

## Methods

### Plant material and growth conditions

Rice (*Oryza sativa* L. ssp. Japonica cv. TNG67) seeds were germinated in darkness at 30 °C for 3 days. The etiolated rice seedlings were cultured in hydroponic solutions [62] containing modified nitrogen sources, with (+N) or without (−N) 1.43 mM NH_4_NO_3_, or supplemented with 0.1-10 mM glutamate, in a controlled growth chamber at 30 °C under a 12-h light/12-h dark photoperiod with 200 μmol photons m^−2^ s^−1^ light intensity and 70% relative humidity for 2 weeks. The hydroponic solution was renewed every 3 days in all experiments. The hydroponic solution recommended by The International Rice Research Institute contains 1.43 mM NH_4_NO_3_ [62], which was used as a control (+N) in all experiments conducted in this study.

### Measurement of chlorophyll content

The Chlorophyll Content Meter (CCM-300, Opti-sciences, NH, USA) was used to measure the amount of chlorophyll in leaves of 17-day-old rice seedlings grown in hydroponic solutions + N, −N, or supplemented with 0.1-10 mM glutamate as the sole nitrogen source.

### RNA isolation and microarray analysis

Total RNA extracted from roots and shoots of 17-day-old rice seedlings grown in hydroponic solution -N or +2.5 mM glutamate for 30 min was used for microarray analysis with the GeneChip Rice Genome Array (Affymetrix, Santa Clara, CA, USA). The method for total RNA isolation was as described previously [63]. RNA samples from two biological repeats were used for the microarray experiment conducted by the Affymetrix Gene Expression Service Lab at Academia Sinica, Taipei, Taiwan (http://ipmb.sinica.edu.tw/affy/). Target preparation, hybridization, washes, staining, array scanning, and data analysis were performed as described [33]. Two-fold cutoff and a *P*-value less than 0.05 were applied to select for up- and down-regulated genes after 2.5 mM Glu treatment for 30 min. AgriGO [64] was used to perform the gene ontology (GO) analysis of 122 glutamate up-regulated genes compared with the genome-wide background with an adjusted *p*-value (False Discovery Rate, FDR) cutoff of 0.05. The GO categories consisting of three structured networks, e.g. biological process, cellular component and molecular function, of defined terms were derived from Gene Ontology (www.geneontology.org). Kyoto Encyclopedia of Genes and Genomes (KEGG) analysis of 122 glutamate up-regulated genes was performed using BlastKOALA (http://www.kegg.jp/blastkoala/). A web-based program EXPath (http://expath.itps.ncku.edu.tw/enrichment/rice/enrichment_analysis.php) was used to analyze KEGG pathway enrichment with the thresholds of *P*-value < 0.05 [65].

### Quantitative RT-PCR analysis of glutamate-responsive genes

To examine the effect of different glutamate concentrations on the expression of glutamate-responsive genes, 17-day-old rice seedlings grown in –N hydroponics were transferred to solutions containing 0–5 mM glutamate for 30 min. For the time course experiment with different nitrogen treatments, 17-day-old rice seedlings grown in –N hydroponics were transferred to solutions containing 2.5 mM glutamate, glutamine, or 1.43 mM NH_4_NO_3_ for 0–24 h. Total RNA extracted from roots of glutamate-treated rice seedlings was digested with DNase I and used for qRT-PCR analysis. All of the quantifications were normalized to the nuclear gene *UBC3* (*Os02g0634800*). The primers used for qRT-PCR analysis are listed in Additional file 1: Table S6. The qRT-PCRs were performed in triplicate for each sample in three independent experiments.

### Amino acid and GABA analysis

For amino acid and GABA analysis, 17-day-old rice seedlings grown in -N hydroponics were transferred to fresh -N or -N supplemented with 2.5 mM glutamate for 30 min or the indicated time. Roots and shoots were harvested separately amino acid extraction. The method for amino acid extraction was described previously [33]. Amino acid samples from four biological repeats were analyzed using the Waters Acquity UPLC system equipped with a Waters AccQ•Tag Ultra column (2.1 mm × 10 mm, 1.7 μm particles) as described [33].

## Additional file

Additional file 1: Figure S1. Glutamate is a precursor for many important molecules in plants. **Figure S2.** Amino acid contents of 17-day-old rice seedlings. **Figure S3.** Regulation of glutamate-responsive genes by different concentrations of glutamate. **Figure S4.** Effects of different nitrogen treatments on the expression of glutamate-responsive genes. **Table S1.** Effects of exogenous glutamate treatment on endogenous amino acid content in rice roots. **Table S2.** List of glutamate up-regulated genes in representative functional categories derived from gene ontology (GO) enrichment analysis. **Table S3.** KEGG analysis of glutamate up-regulated genes in rice roots. **Table S4.** KEGG pathway enrichment analysis of glutamate up-regulated genes in rice root. **Table S5.** Effects of glutamate on the expression of glutamate receptor genes in rice roots. **Table S6.** Sequences of primers used for quantitative RT-PCR analysis. (PDF 2009 kb)

## Abbreviations

GABA: γ-aminobutyrate
GDC: Glutamate decarboxylase
GDH: Glutamate dehydrogenase
GLR: Glutamate receptor
GO: Gene ontology
GOGAT: Glutamine-oxoglutarate aminotransferase
GS: Glutamine synthetase
GSH: Glutathione
HI-LOX: Herbivore induced 13-lipoxygenase
KEGG: Kyoto encyclopedia of genes and genomes
KO: KEGG orthology
qRT-PCR: Quantitative reverse transcription-polymerase chain reaction
WAK: Wall associated kinase
