# Supplementary material for: Exogenous glutamate rapidly induces the expression of genes involved in metabolism and defense responses in rice roots
Source: BMC Genomics. 2017 Feb 17;18:186. doi: 10.1186/s12864-017-3588-7 (PMC5316172; doi:10.1186/s12864-017-3588-7)
Supplement: Additional file 1: Figure S1. — Glutamate is a precursor for many important molecules in plants. Figure S2. Amino acid contents of 17-day-old rice seedlings. Figure S3. Regulation of glutamate-responsive genes by different concentrations of glutamate. Figure S4. Effects of different nitrogen treatments on the expression of glutamate-responsive genes. Table S1. Effects of exogenous glutamate treatment on endogenous amino acid content in rice roots. Table S2. List of glutamate up-regulated genes in representative functional categories derived from gene ontology (GO) enrichment analysis. Table S3. KEGG analysis of glutamate up-regulated genes in rice roots. Table S4. KEGG pathway enrichment analysis of glutamate up-regulated genes in rice root. Table S5. Effects of glutamate on the expression of glutamate receptor genes in rice roots. Table S6. Sequences of primers used for quantitative RT-PCR analysis. (PDF 2009 kb) [file 12864_2017_3588_MOESM1_ESM.pdf]

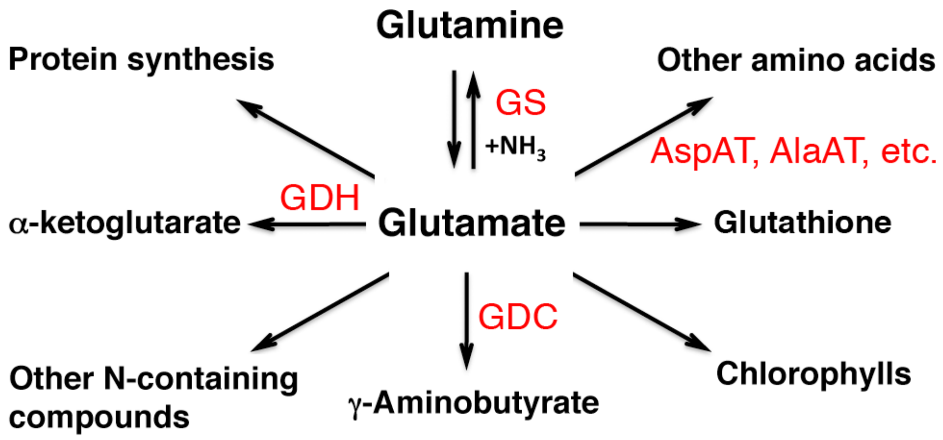

Figure S1. Glutamate is a precursor for many important molecules in plants. Feeding of glutamate to rice seedlings grown in hydroponics significantly increased the endogenous levels of glutamine, aspartate, and alanine in the roots. The conversions of glutamate to glutamine, aspartate, and alanine are catalyzed by glutamine synthetase (GS), aspartate aminotransferase (AspAT), and alanine aminotransferase (AlaAT), respectively. Exogenous glutamate treatment also rapidly induced the expression of *glutamate dehydrogenase* (*GDH*) and *glutamate decarboxylase* (*GDC*) genes in rice roots.

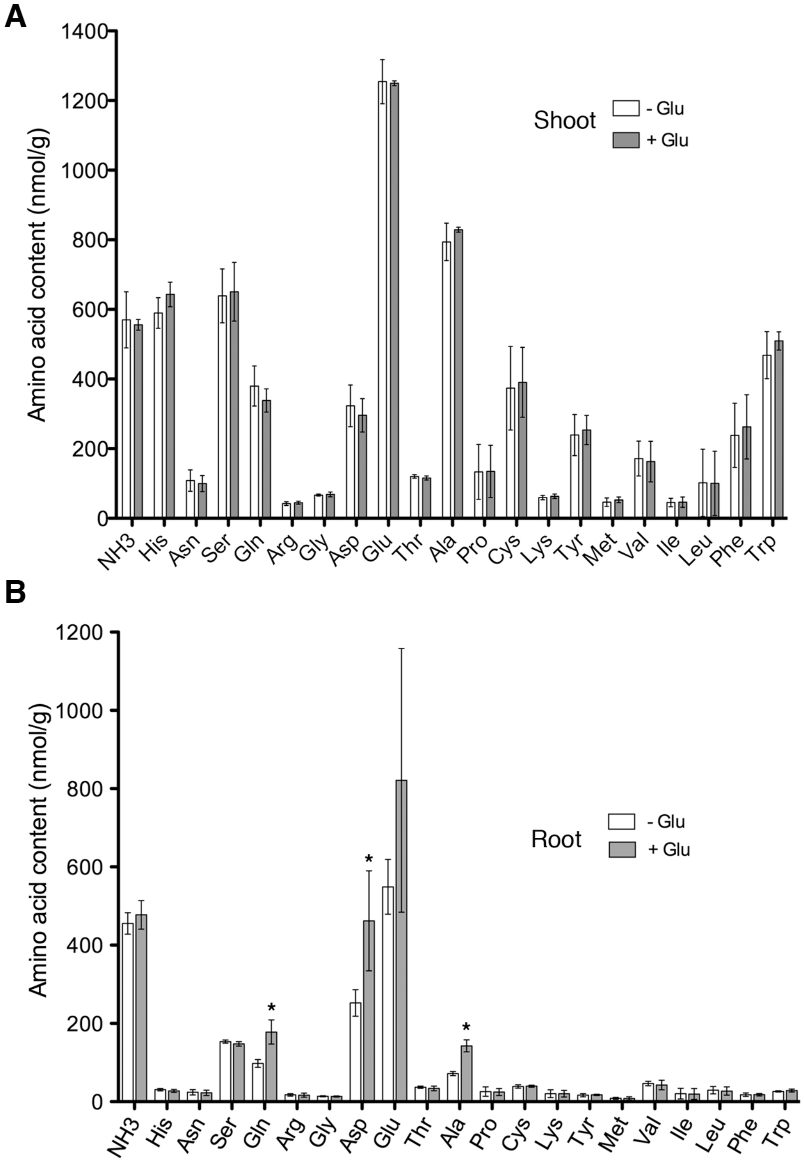

Figure S2. Amino acid contents of 17-day-old rice seedlings. Shoots **(A)** and roots **(B)** from nitrogen-starved (- Glu) or 2.5 mM glutamate-treated (+ Glu, 30 min) rice seedlings were used for amino acid analysis. Results are derived from four biological repeats. Asterisk indicates $p < 0.05$ after analysis with Student's *t*-test.

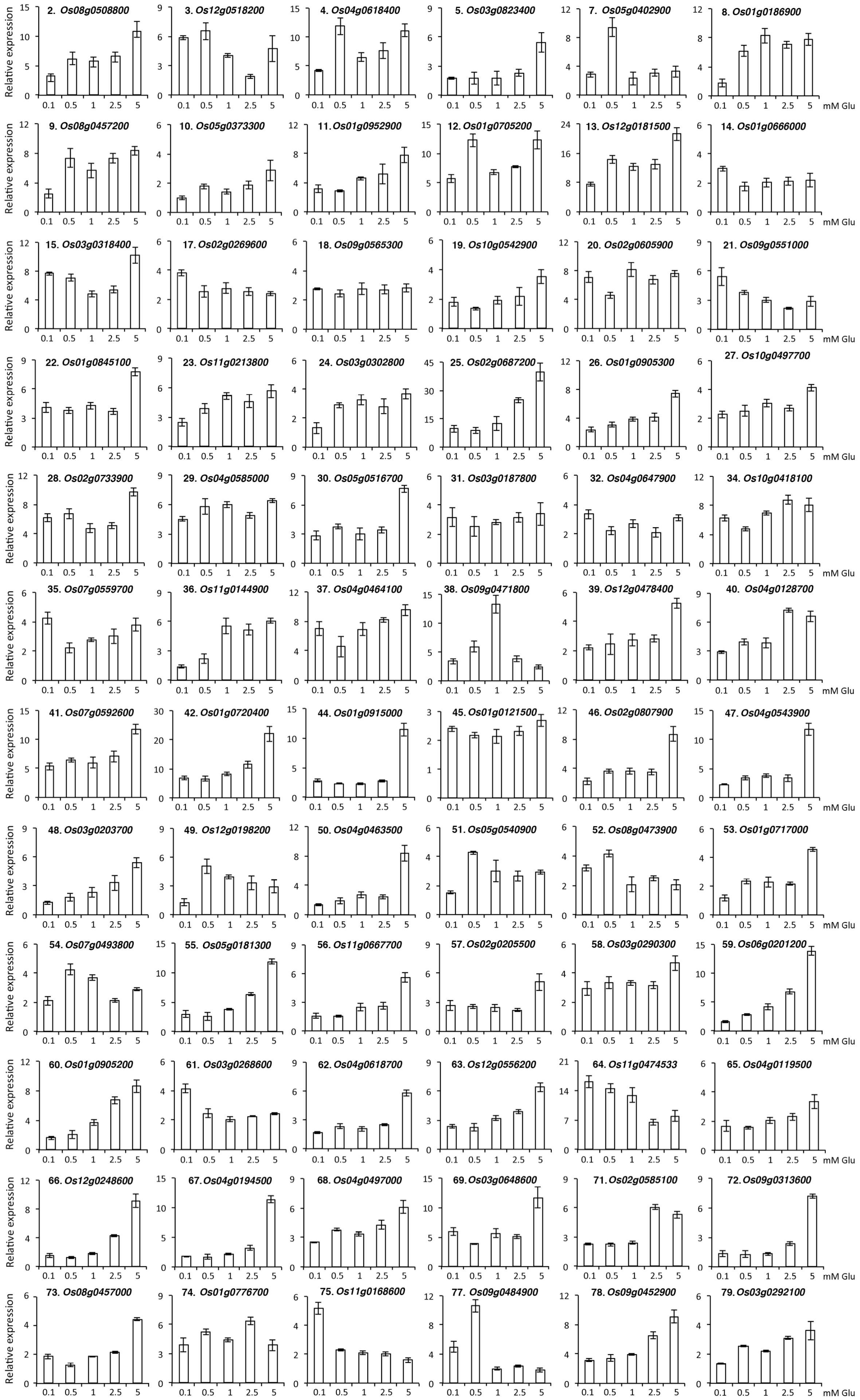
Relative expression
mM Glu
0.1 0.5 1 2.5 5
2. Os08g0508800
3. Os12g0518200
4. Os04g0618400
5. Os03g0823400
7. Os05g0402900
8. Os01g0186900
9. Os08g0457200
10. Os05g0373300
11. Os01g0952900
12. Os01g0705200
13. Os12g0181500
14. Os01g0666000
15. Os03g0318400
17. Os02g0269600
18. Os09g0565300
19. Os10g0542900
20. Os02g0605900
21. Os09g0551000
22. Os01g0845100
23. Os11g0213800
24. Os03g0302800
25. Os02g0687200
26. Os01g0905300
27. Os10g0497700
28. Os02g0733900
29. Os04g0585000
30. Os05g0516700
31. Os03g0187800
32. Os04g0647900
34. Os10g0418100
35. Os07g0559700
36. Os11g0144900
37. Os04g0464100
38. Os09g0471800
39. Os12g0478400
40. Os04g0128700
41. Os07g0592600
42. Os01g0720400
44. Os01g0915000
45. Os01g0121500
46. Os02g0807900
47. Os04g0543900
48. Os03g0203700
49. Os12g0198200
50. Os04g0463500
51. Os05g0540900
52. Os08g0473900
53. Os01g0717000
54. Os07g0493800
55. Os05g0181300
56. Os11g0667700
57. Os02g0205500
58. Os03g0290300
59. Os06g0201200
60. Os01g0905200
61. Os03g0268600
62. Os04g0618700
63. Os12g0556200
64. Os11g0474533
65. Os04g0119500
66. Os12g0248600
67. Os04g0194500
68. Os04g0497000
69. Os03g0648600
71. Os02g0585100
72. Os09g0313600
73. Os08g0457000
74. Os01g0776700
75. Os11g0168600
77. Os09g0484900
78. Os09g0452900
79. Os03g0292100

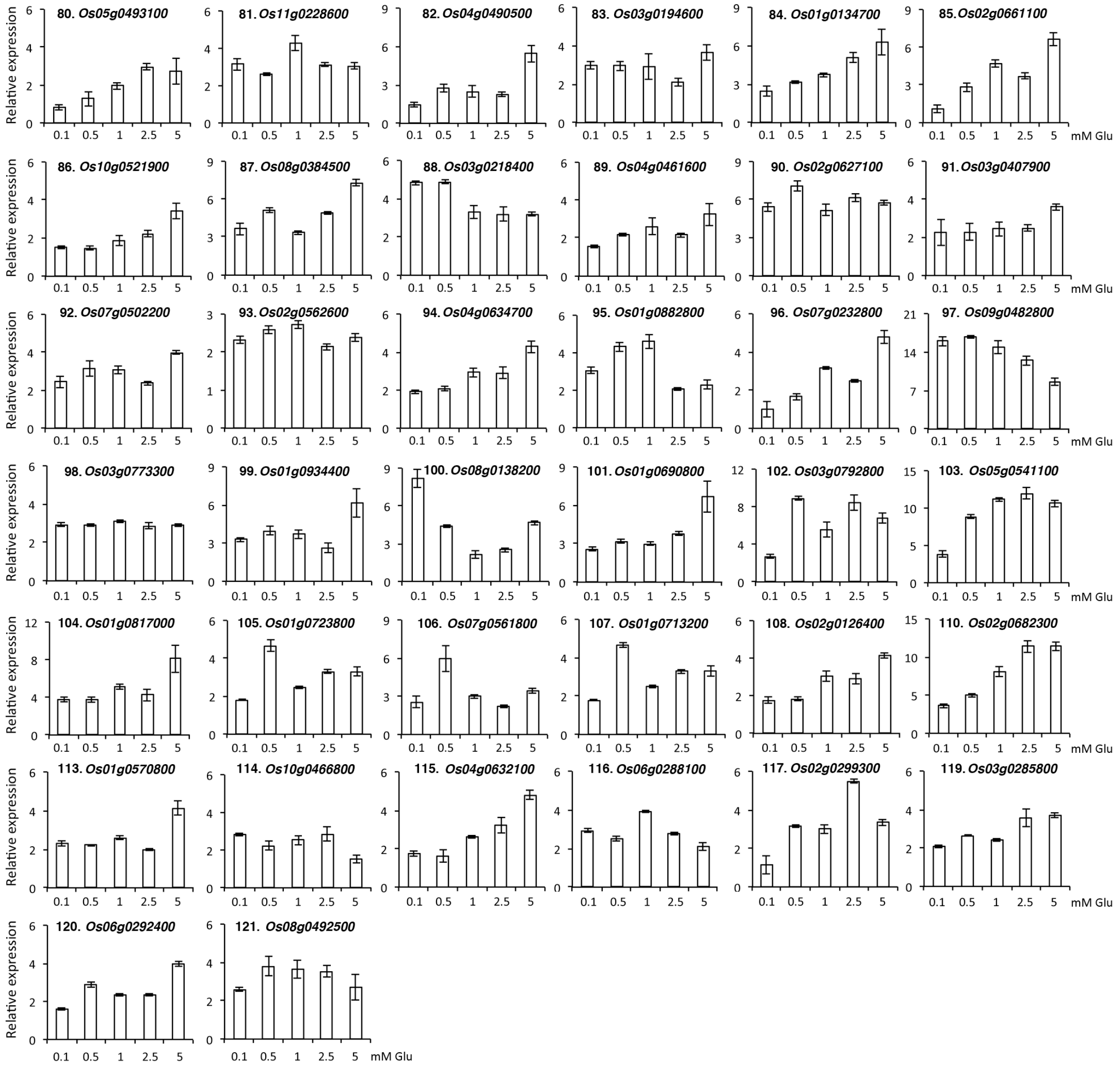

Figure S3. Regulation of glutamate-responsive genes by different concentrations of glutamate. Total RNA extracted from roots of 17-day-old rice seedlings treated with 0-5 mM glutamate for 30 min was used for qRT-PCR to analyze the expression of 110 glutamate-responsive genes. The number of each gene corresponds to the number in Table 1. Relative expression indicates the fold-change of each gene as compared to that of control (0 mM). The expression of *late embryogenesis abundant protein* (12. *Os01g0705200*), *amino acid permease 3* (13. *Os12g0181500*), *unknown* (25. *Os02g0687200*), *OsGH3.8* (41. *Os07g0592600*), *inorganic pyrophosphatase 1* (42. *Os01g0720400*), *unknown* (64. *Os11g0474533*), *phenylalanine ammonia-lyase* (90. *Os02g0627100*), and *EF-hand domain containing protein* (97. *Os09g0482800*) was very sensitive to glutamate induction (fold-change > 5). The result of *GDC1* (1. *Os03g0236200*) is shown in Fig. 7A.

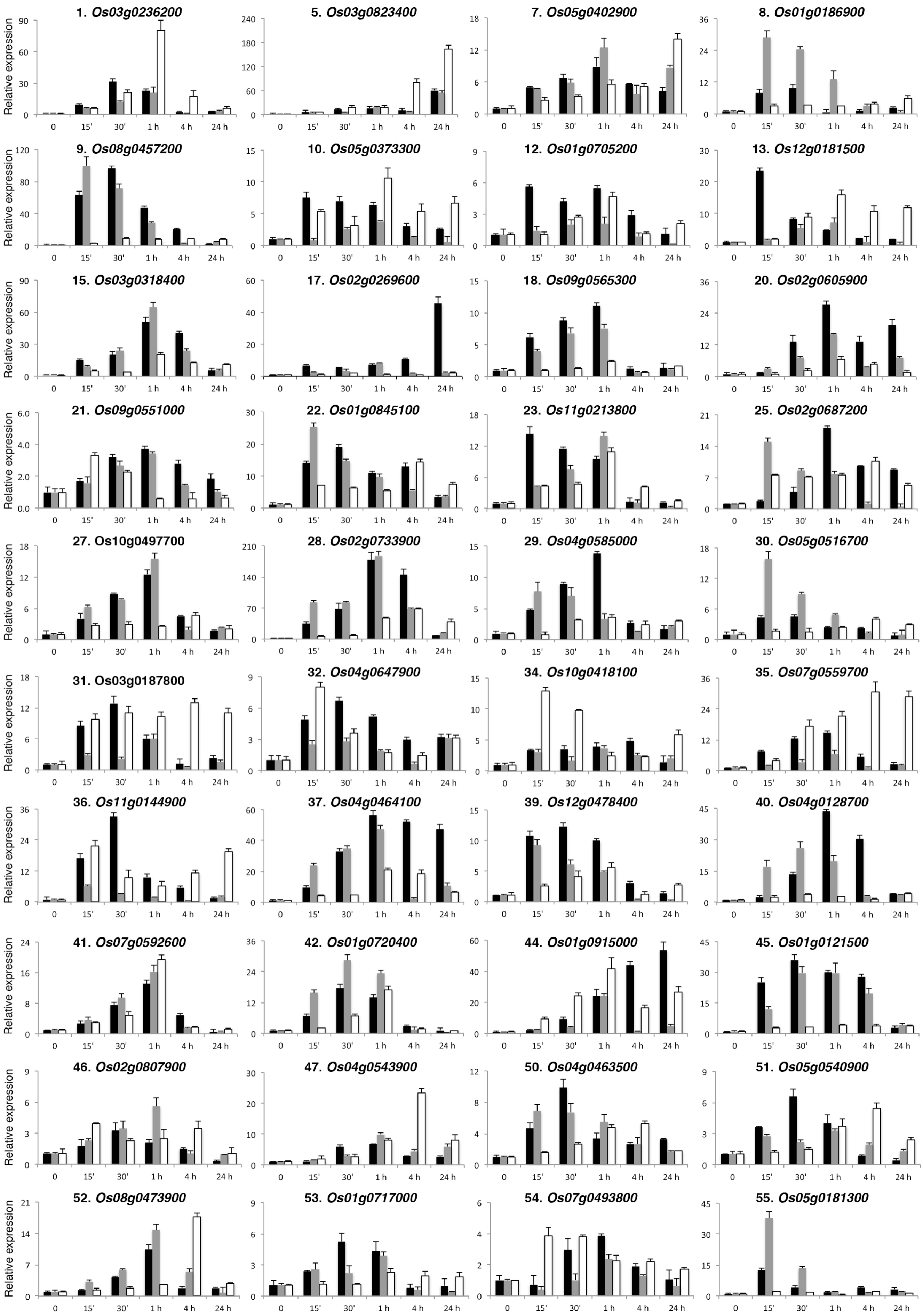
1. Os03g0236200
5. Os03g0823400
7. Os05g0402900
8. Os01g0186900
9. Os08g0457200
10. Os05g0373300
12. Os01g0705200
13. Os12g0181500
15. Os03g0318400
17. Os02g0269600
18. Os09g0565300
20. Os02g0605900
21. Os09g0551000
22. Os01g0845100
23. Os11g0213800
25. Os02g0687200
27. Os10g0497700
28. Os02g0733900
29. Os04g0585000
30. Os05g0516700
31. Os03g0187800
32. Os04g0647900
34. Os10g0418100
35. Os07g0559700
36. Os11g0144900
37. Os04g0464100
39. Os12g0478400
40. Os04g0128700
41. Os07g0592600
42. Os01g0720400
44. Os01g0915000
45. Os01g0121500
46. Os02g0807900
47. Os04g0543900
50. Os04g0463500
51. Os05g0540900
52. Os08g0473900
53. Os01g0717000
54. Os07g0493800
55. Os05g0181300
Relative expression
0
15'
30'
1 h
4 h
24 h

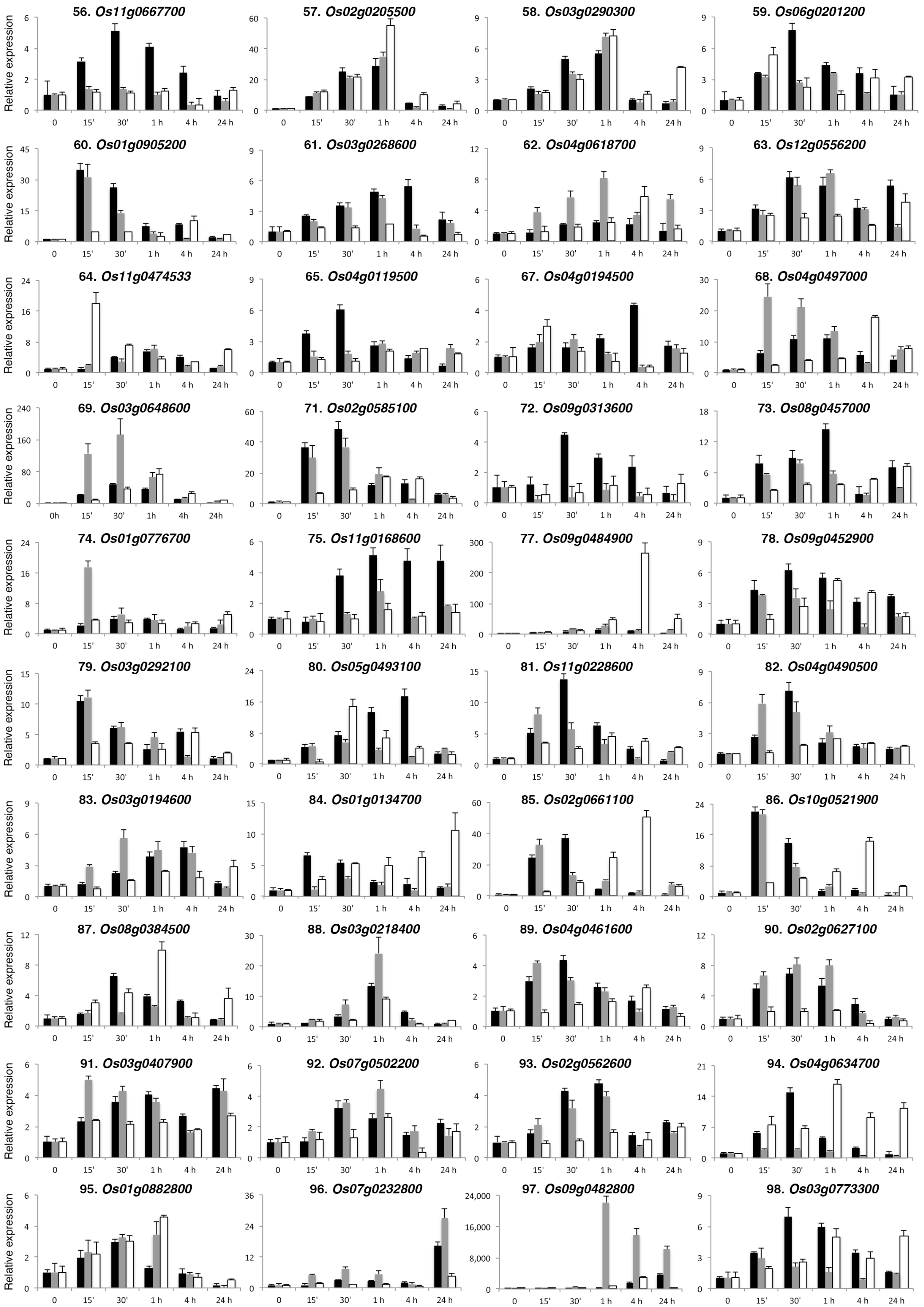
56. Os11g0667700
57. Os02g0205500
58. Os03g0290300
59. Os06g0201200
60. Os01g0905200
61. Os03g0268600
62. Os04g0618700
63. Os12g0556200
64. Os11g0474533
65. Os04g0119500
67. Os04g0194500
68. Os04g0497000
69. Os03g0648600
71. Os02g0585100
72. Os09g0313600
73. Os08g0457000
74. Os01g0776700
75. Os11g0168600
77. Os09g0484900
78. Os09g0452900
79. Os03g0292100
80. Os05g0493100
81. Os11g0228600
82. Os04g0490500
83. Os03g0194600
84. Os01g0134700
85. Os02g0661100
86. Os10g0521900
87. Os08g0384500
88. Os03g0218400
89. Os04g0461600
90. Os02g0627100
91. Os03g0407900
92. Os07g0502200
93. Os02g0562600
94. Os04g0634700
95. Os01g0882800
96. Os07g0232800
97. Os09g0482800
98. Os03g0773300
Relative expression
0
15'
30'
1 h
4 h
24 h
0h
1h
4h
24h

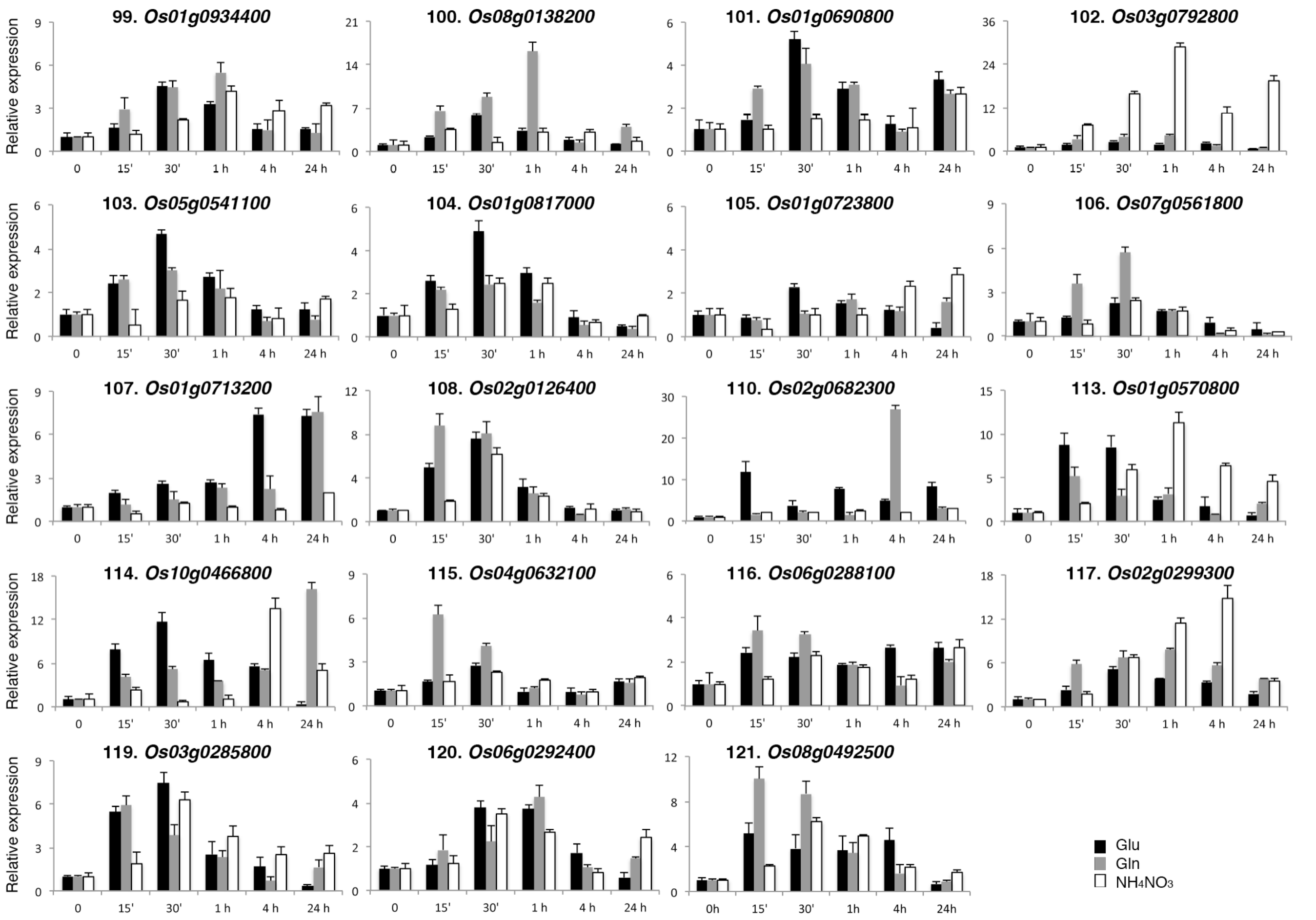

Figure S4. Effects of different nitrogen treatments on the expression of glutamate-responsive genes. Total RNA extracted from roots of 17-day-old rice seedlings treated with 2.5 mM glutamate, glutamine, or 1.43 mM NH_4NO_3 for 0, 15', 30', 1, 4, and 24 h was used for qRT-PCR to analyze the expression of glutamate-responsive genes. The number of each gene corresponds to the number in Table 1. Relative expression indicates the fold-change of each gene as compared to that of control (0 h).

Table S1. Effects of exogenous glutamate treatment on endogenous amino acid content (nmole/g FW) in rice roots

	0	15'	30'	1 h	4 h	8 h	16 h	24 h
Glu	286.2±14.6	335.5±35.2	336.1±36.0	449.6±51.4	708.7±31.8	664.5±44.2	744.6±14.5	734.9±58.8
Gln	88.1±10.3	128.1±17.4	146.4±27.3	289.1±42.2	343.2±51.8	801.5±80.1	861.2±96.9	641.8±254.1
Asp	182.3±20.1	287.7±38.0	301.5±58.7	339.1±28.5	571.7±46.6	437.8±35.1	400.3±45.1	492.6±83.4
Asn	48.2±5.6	43.4±7.9	40.6±7.4	45.6±5.4	156.3±7.6	466.9±33.2	617.3±35.4	674.4±212.9
Ala	82.8±9.3	116.2±15.4	88.5±56.0	155.8±31.4	194.5±24.5	227.3±20.0	604.8±29.6	561.2±143.4
Ser	122.9±8.7	113.6±5.8	102.3±14.5	113.6±11.3	143.5±14.9	201.7±16.0	232.8±28.9	292.7±47.1

Table S2. List of glutamate up-regulated genes in representative functional categories derived from gene ontology (GO) enrichment analysis.

GO term	*p*-value	Genes*
metabolic process	1.10E-05	*Os03g0236200* (1), *Os09g0401000* (6), *Os05g0402900* (7), *Os01g0186900* (8), *Os03g0318400* (15), *Os04g0301500* (16), *Os02g0269600* (17), *Os09g0565300* (18), *Os10g054290*0 (19), *Os02g0605900* (20), *Os09g0551000* (21), *Os10g0418100* (34), *Os09g0471800* (38), *Os01g0720400* (42), *Os02g0807900* (46), *Os04g0543900* (47), *Os03g0203700* (48), *Os12g0198200* (49), *Os04g0463500* (50), *Os07g0493800* (54), *Os11g0667700* (56), *Os02g0205500* (57), *Os03g0290300* (58), *Os01g0905200* (60), *Os03g0268600* (61), *Os04g0618700* (62), *Os04g0497000* (68), *Os11g0154500* (70), *Os11g0168600* (75), *Os09g0455300* (76), *Os09g0452900* (78), *Os03g0292100* (79), *Os05g0493100* (80), *Os04g0490500* (82), *Os02g0661100* (85), *Os02g0627100* (90), *Os04g0634700* (94), *Os03g0773300* (98), *Os01g0934400* (99), *Os01g0690800* (101), *Os03g0792800* (102), *Os01g0713200* (107), *Os02g012640*0 (108), *Os07g0119300* (109), *Os11g0184900* (112), *Os04g0632100* (115), *Os06g0288100* (116), *Os03g0285800* (119)
protein kinase activity	4.00E-05	*Os09g0551000* (21), *Os10g0418100* (34), *Os02g0807900* (46), *Os07g0493800* (54), *Os11g0667700* (56), *Os04g0618700* (62), *Os11g0168600* (75), *Os05g0493100* (80), *Os04g0490500* (82), *Os03g0773300* (98), *Os01g0690800* (101), *Os02g0126400* (108), *Os04g0632100* (115), *Os06g0288100* (116), *Os03g0285800* (119)
transporter activity	0.0054	*Os10g0418100* (34), *Os07g0559700* (35), *Os03g0203700* (48), *Os01g0905200* (60), *Os09g0484900* (77), *Os03g0218400* (88), *Os07g0502200* (92), *Os07g0232800* (96), *Os01g0723800* (105)
ATP binding	0.00072	*Os09g0551000* (21), *Os11g0213800* (23), *Os10g0418100* (34), *Os02g0807900* (46), *Os03g0203700* (48), *Os07g0493800* (54), *Os04g0618700* (62), *Os11g0667700* (56), *Os04g0194500* (67), *Os11g0168600* (75), *Os05g0493100* (80), *Os04g0490500* (82), *Os03g0773300* (98), *Os01g0690800* (101), *Os01g0723800* (105), *Os02g0126400* (108), *Os03g0285800* (119), *Os04g0632100* (115), *Os06g0288100* (116)
Calcium ion binding	9.90E-05	*Os10g0418100* (34), *Os09g0471800* (38), *Os03g0203700* (48), *Os08g0473900* (52), *Os09g0482800* (97), *Os01g0934400* (99), *Os02g0126400* (108)

* The number in parentheses corresponds to the number listed in Table 1.

Table S3. KEGG analysis of glutamate up-regulated genes in rice roots.

KO number	Gene ID*	Gene annotation	KEGG pathway
K01580	Os03g0236200 (1)	Glutamate decarboxylase [EC:4.1.1.15]	Alanine, aspartate and glutamate metabolism [ko00250], beta-Alanine metabolism [ko00410], Taurine and hypotaurine metabolism [ko00430], Butanoate metabolism [ko00650]
K00454	Os08g0508800 (2)	Lipoxygenase [EC:1.13.11.12]	Linoleic acid metabolism [ko00591], alpha-Linolenic acid metabolism [ko00592]
K09422	Os09g0401000 (6)	Myb proto-oncogene protein, plant	Transcription factors [BR: ko03000]
K20547	Os10g0542900 (19)	Chitinase [EC:3.2.1.14]	Amino sugar and nucleotide sugar metabolism [ko00520], MAPK signaling pathway - plant [ko04016]
K01183	Os02g0605900 (20)	Chitinase [EC:3.2.1.14]	Amino sugar and nucleotide sugar metabolism [ko00520]
K07195	Os01g0905300 (26)	Exocyst complex component 7	Insulin signaling pathway [ko04910]
K01537	Os10g0418100 (34)	Calcium-transporting ATPase [EC:3.6.3.8]	Enzymes [BR: ko01000]
K14487	Os07g0592600 (41)	GH3; auxin responsive GH3 gene family	Plant hormone signal transduction [ko04075]
K13248	Os01g0720400 (42)	PDXP; pyridoxal phosphatase [EC:3.1.3.74]	Vitamin B6 metabolism [ko00750]
K00261	Os04g0543900 (47)	Glutamate dehydrogenase [EC:1.4.1.3]	Arginine biosynthesis [ko00220], Alanine, aspartate and glutamate metabolism [ko00250], D-Glutamine and D-glutamate metabolism [ko00471], Nitrogen metabolism [ko00910], Carbon metabolism [ko01200]
K01537	Os03g0203700 (48)	Calcium-transporting ATPase [EC:3.6.3.8]	Enzymes [BR: ko01000]
K17506	Os12g0198200 (49)	PP2C; protein phosphatase [EC:3.1.3.16]	Enzymes [BR: ko01000]
K01658	Os04g0463500 (50)	Anthranilate synthase beta [EC:4.1.3.27]	Phenylalanine, tyrosine and tryptophan biosynthesis [ko00400], Biosynthesis of amino acids [ko01230]
K01176	Os08g0473900 (52)	Alpha-amylase [EC:3.2.1.1]	Starch and sucrose metabolism [ko00500], Carbohydrate digestion and absorption [ko04973]
K14156	Os01g0717000 (53)	Choline kinase [EC:2.7.1.32 2.7.1.82]	Glycerophospholipid metabolism [ko00564]
K15397	Os02g0205500 (57)	3-ketoacyl-CoA synthase [EC:2.3.1.199]	Fatty acid elongation [ko00062]
K10257	Os03g0290300 (58)	Omega-3 fatty acid desaturase [EC:1.14.19.25 1.14.19.35 1.14.19.36]	Enzymes [BR: ko01000]
K07195	Os01g0905200 (60)	Exocyst complex component 7	Insulin signaling pathway [ko04910]
K14497	Os03g0268600 (61)	PP2C; protein phosphatase 2C [EC:3.1.3.16]	MAPK signaling pathway – plant [ko04016], Plant hormone signal transduction [ko04075]
K13420	Os04g0618700 (62)	FLS2; LRR receptor-like serine/threonine-protein kinase [EC:2.7.11.1]	MAPK signaling pathway - plant [ko04016], Plant-pathogen interaction [ko04626]
K14445	Os09g0484900 (77)	Sodium-dependent dicarboxylate transporter	Transporters [BR: ko02000]
K20855	Os09g0452900 (78)	Beta-1,3-galactosyltransferase [EC:2.4.1.-]	Enzymes [BR: ko01000]
K01087	Os02g0661100 (85)	Trehalose 6-phosphate phosphatase [EC:3.1.3.12]	Starch and sucrose metabolism [ko00500]
K10775	Os02g0627100 (90)	Phenylalanine ammonia-lyase [EC:4.3.1.24]	Phenylalanine metabolism [ko00360], Phenylpropanoid biosynthesis [ko00940]
K03327	Os07g0502200 (92)	MATE family protein	Transporters [BR: ko02000]
K00901	Os04g0634700 (94)	Diacylglycerol kinase [EC:2.7.1.107]	Glycerolipid metabolism [ko00561], Glycerophospholipid metabolism [ko00564], Phosphatidylinositol signaling system [ko04070],

			Phospholipase D signaling pathway [ko04072]
K14709	Os07g0232800 (96)	Zinc transporter	Transporters [BR: ko02000]
K20858	Os01g0817000 (104)	Calcium uniporter protein	Calcium signaling pathway [ko04020], NOD-like receptor signaling pathway [ko04621]
K05658	Os01g0723800 (105)	ATP-binding cassette, subfamily B [EC:3.6.3.44]	ABC transporters [ko02010]
K13412	Os02g0126400 (108)	Calcium-dependent protein kinase [EC:2.7.11.1]	Plant-pathogen interaction [ko04626]
K16281	Os02g0682300 (110)	RING-H2 zinc finger protein RHA1	Ubiquitin system [BR: ko04121]
K20536	Os03g0285800 (119)	Mitogen-activated protein kinase [EC:2.7.11.24]	MAPK signaling pathway - plant [ko04016]
K16285	Os08g0492500 (121)	XERICO; RING/U-box domain-containing protein [EC:2.3.2.27]	Enzymes [BR: ko01000]

* The number in parentheses corresponds to the number listed in Table 1.

Table S4. KEGG pathway enrichment analysis of glutamate up-regulated genes in rice root.

Pathway ID	Pathway name	Hit number (Query)	*p*-value
ko00564	Glycerophospholipid metabolism	2	0.03
ko02010	ABC transporters	1	0.04

Table S5. Effects of glutamate on the expression of glutamate receptor genes in rice roots.

Gene	Locus ID		Fold change (+ Glu/- N)
OsGLR1.1	Os09g0431100	LOC_Os09g26144	0.76
OsGLR1.2	Os09g0431200	LOC_Os09g26160	1.39
OsGLR1.3	Os06g0190800	LOC_Os06g09130	1.07
OsGLR1.4	Os02g0787600	LOC_Os02g54640	1.18
OsGLR2.1	Os09g0429200	LOC_Os09g25980	0.61
OsGLR2.2	Os09g0429000	LOC_Os09g25960	0.82
OsGLR2.3	Os09g0429400	LOC_Os09g25990	0.82
OsGLR2.4	Os09g0429500	LOC_Os09g26000	0.82
OsGLR3.1	Os04g0585200	LOC_Os04g49570	1.06
OsGLR3.2	Os02g0117500	LOC_Os02g02540	1.60
OsGLR3.3	Os06g0680500	LOC_Os06g46670	0.99
OsGLR3.4	Os07g0103100	LOC_Os07g01310	1.13
OsGLR3.5	Os06g0155000	LOC_Os06g06130	1.13

Table S6. Sequences of primers used for quantitative RT-PCR analysis

No.	Locus ID	Primer sequence 5'→3'
1	Os03g0236200	AGGAGCTCATCTTCCACATCAA / GATGTTCCTGTACCCCTCGAA
2	Os08g0508800	ACGCCTAGCAAACGCATCTTC / CTGTCCCGGCAAGTAAGTCTTG
3	Os12g0518200	TTGCTTGCCATCCTATACTCGG / TAGGCACCACGTTTGCAGGTA
4	Os04g0618400	CCAAAGCAGAGAGTCCTTGAGA / GACCGATGGTTTTCACGTAAAG
5	Os03g0823400	AAAACGAAAGCGAGAGATGAGG / ACAAAGGCCAAGAACAGAGCC
6	Os09g0401000	AGTTCGAGGATCCGTTCAACG / GTGGTGGTGATCAAACAACGC
7	Os05g0402900	GTCACATGATGGAGGACAACCT / CATCTAGCCGAGGCGAAAGTT
8	Os01g0186900	ATCTACACCACCCACATCCCCA / AGTAGGAGGCGACGTTGGACTT
9	Os08g0457200	TGCCTCAAAACGTGGTCCAT / ATGCAAAGACGAGGACAGGG
10	Os05g0373300	TACATTGACCCCTCTGGCAGAC/ TTCGCCAACCCCTAG TATTGC
11	Os01g0952900	TTCTTCCATCTGCAAGCTCGA/ TGCCCATGGCAACTGATGT
12	Os01g0705200	GGCGATAACAAGAACAACGCC / AGTCACTCCCAGTTCCAGGCTT
13	Os12g0181500	TCATGTCCTTCACCTACTCCGG / TGGAGATTGTCTGGACGATGC
14	Os01g0666000	CCGGCTCCTTCAGATGAGAAA / CGGTGATGTACCTAAAGTGCGC
15	Os03g0318400	TTGCCATTATTGAAGGCGGA / TTCTGCTGCTGGAAATTGCC
16	Os04g0301500	GTCATTTTTGCGACACCCCA / CCTCGCAAGAAAACAACCACC
17	Os02g0269600	TCGACGACGCCATAAACGAT / GCCCTCCTAACGACAGTGACAA
18	Os09g0565300	GCAAGAGGATCAGTGATCTGGC / GGAGGAAGAGGCATCCTTGAAG
19	Os10g0542900	TATGGACGTGGCCCAATTCA / CGGCTGCTTGGTAGTTCGATT
20	Os02g0605900	ACGCCAACCACGAGACCATAA / GTCCGTCGATCTCCTCGATGTA
21	Os09g0551000	GAAGGAAGGCAAGGCGAAAA / ATCCAATGAACTGCTCTCCGC
22	Os01g0845100	CTGAATCCATCCATTGAACCGT / GCAAAAGTTTCCCGCGTGT
23	Os11g0213800	CCATCGTAACCACCCGAGATAA / CGGTTCAGTGGTGCAAATCTTC
24	Os03g0302800	CATGCAGTTGCTTTGGCTCA / CCATGCGGATACACAGCAGTAC
25	Os02g0687200	TCAACCGGTAAGGCATTGTAGC / GGTCAGGATGGCCAAGATCAT
26	Os01g0905300	GAAAGTTGCCGCCAAGTTCA / TGACTCTCGTACGCTTCGTCAA
27	Os10g0497700	TGACAAAACTGTTGACTGCCCA / TTTTTCATTTCGGCAGCCAC
28	Os02g0733900	ACTGATTGAGCAGCCGAACA / CGCAGTACAGCCAAAGAATGAT
29	Os04g0585000	GCTGGAAATGGATAGCATTTGG / GCAAACCAGGATCTCTGACTCG
30	Os05g0516700	GGACGCTCGCATCTATGGTTAA / AAATGCGAGTATCCGTTAGGCA
31	Os03g0187800	TCATGGCCACCATTACTGCTG / TTGCTGGTCTCTTCCATGGCT
32	Os04g0647900	GGACCTTTCCCACAATGAACTG / TTTGGTCAGCTGCCATGGA
33	Os01g0705700	TGCGTACGGTATAGCTGCCAA/ TGAAGTCACAAGGAGTTGCACC
34	Os10g0418100	TCATCCAGTTCCAGCTCACCGT / GACACGAAGTTGATGACGAGCG
35	Os07g0559700	GATCACCATTGGCATCCTGTG / GATCTTGGCAGTCCCGTAGTTG
36	Os11g0144900	ACGTATGCCTGAATGGCTCAAG / CCTCGACATGCTTCTCCAACTC
37	Os04g0464100	GTGAAGGACAAGAAGCCGGAG / GATTTACATGATGGAGCAGGGGCT
38	Os09g0471800	TGATCGGATGCAAGACTCTCG / GCTGACGAACACCCGATTACAT
39	Os12g0478400	AAATCCCTACGTTGCTGGTGG / TAATCATCCGAGCGAGCACAC
40	Os04g0128700	TAGTTGCTCCCGTGAAACCGT / TGAAAAGCCAGTGTCACCGAC
41	Os07g0592600	CGAATAGTCGGTCAAATCCCC / CAGATTCTTGGACACGGTCCA
42	Os01g0720400	TGACTGATTTTTGCGCTACGC / TGAGCCTCTACTAATCGCAGGC
43	Os02g0764700	CAGCCACGTCCACTCCTCCA/ ATGATCTCCCGTAGGCCGTC
44	Os01g0915000	CAGACACCTCAAATCCACGCA / GGATGTAGACGGCCATTTTCG
45	Os01g0121500	AGCATTCGCTAGCTGCACAATT / GCCGGTTGCATTTTTGGATT
46	Os02g0807900	TCGTGCTGGAGCTTCTAACCA / TCTGGCCCGTCTAGGTACAATG

47	Os04g0543900	AGACCATGGCTTGGATTTTGG / GGTGAATGACCATGGAATTTCG
48	Os03g0203700	GGAATGCGGCATTTTGACTG / CTTGGTTCTGAAATCCGGTCCT
49	Os12g0198200	CATGCAATGGAGGATGGATATG / GACCGATGGTTTTCACGTAAAG
50	Os04g0463500	ATTATCCGTGCTCCTTCTGGTG / GCGAACTGGAGAGCTTTTTCC
51	Os05g0540900	TCGCTGCTCAACTACAAGGAGA / TGTGGCTAGCTACTGCTTGCTG
52	Os08g0473900	TTGTATCCGATTGTAGCGTTCG / CCCGCAATTAACCTAGAGGCT
53	Os01g0717000	TCGACAAACTAGAGGCCGCTT / ACACATGAAACACGACGGCA
54	Os07g0493800	GATGCTCTTTTCGGTAACGCG / CGCAAGCGAAAGGACAATCA
55	Os05g0181300	GTCGTCCAAGCTCGATCAGATC / GCTCCTCTTCCCCAAATCTTTT
56	Os11g0667700	CAAGAACTCTGAAGCCAGCGAG / CCAGCATCACAACGCCAAA
57	Os02g0205500	GGATTTCAACCGACTGAGCTTG / TGCCAATTCCAAAGGAGCAC
58	Os03g0290300	CGGTCATGTCGAGAAAGACGA / TACAGCCTCTCAGACAGCGGTT
59	Os06g0201200	CATTTCTTGATCCCCGTCATG / CGAAAACAATGGCCGTCAC
60	Os01g0905200	ATGTCGATAAGGTCCATGGTCG / ACACCGTCTCGTACCTTCGCTA
61	Os03g0268600	AAACCTCGGACAATGTCAGCG / TTGTCACAATTTCCGGCGC
62	Os04g0618700	TGCATTGCGAACTTTCTCCA / CCTCAGTAAGCTCACACGAACG
63	Os12g0556200	TCATCCCAGGCAGTGCAAAT / TGTGTCACTCGCACAGATCCA
64	Os11g0474533	TCAGGTCACTTGCCTTTGGTTC / CCCTGCATGCTCCTCTAGAATG
65	Os04g0119500	TCTAGTTGGTGCATCCGTCCA / CATTGAGCCATCACCGTTTCTT
66	Os12g0248600	GCCAGCTCGGTTTTGCCTATA / CCCATGCTTTTTCTGACGAAGA
67	Os04g0194500	CGAGAAACGGAAAGCGAGATC / CTGACGTGCCAACTGAATAGCA
68	Os04g0497000	CTGTGCATTGTCCTGATGGCT / CAACGCATTCAGCTAAGCTTGA
69	Os03g0648600	ATCTGCTGTCAGGAATACTCCG / TGGAAGAAATGGCTGCACC
70	Os11g0154500	GAGCCGATTTTGCTTGGAAATC / CCCATGAATCCGATCCCATT
71	Os02g0585100	TGAACTGCTTTCCAAGCTCACC / CGAGGAAATTCGTGTGTGGATC
72	Os09g0313600	ATTTGAGGAGGTAAGACCCCTG / AGCTTTACCATCTGGAGGTTCC
73	Os08g0457000	CAAGCTCGTGAGGCTGAAACA / AGGAGAAATGGCCTTCGGTTC
74	Os01g0776700	ATTCATTTCGTGCTCTCGTGGT / GCCTAGCAATGGCAAACTTTCA
75	Os11g0168600	TGGCCGGTGAAGATCAACAT / TCCCTAGCCCATTTCAGCATC
76	Os09g0455300	TTACCGCATGCTAGCTCGTACG / CGAGTCCAACAAAAGCTCGGA
77	Os09g0484900	GGAACGCCCCTGAAAATTGT / TGGCAGTAGGATGGTGAGAGCT
78	Os09g0452900	ACGTTTCACTTGGCGCATG / CGTCAATGTGCTCCACGTCTAA
79	Os03g0292100	GTGTTGATTCGCAGTGCGAA / TGTCCCACAAACCATCAGAGG
80	Os05g0493100	TGGAAGCGAGCGTAGCAAA / GCCATGTATCCGTAGGTTCCAG
81	Os11g0228600	AATGACCAAGGAAGTCGCGTAA / GCAAGAGCTGCCACATGATTC
82	Os04g0490500	TTGCCCGCGTCATCAACTA / ATCATCAGACCACGCCTTGCT
83	Os03g0194600	GAACCTGAGGAGCACCAAGAA / GCCTACTACGCATCAAGACGTG
84	Os01g0134700	CCACTGCAACGACGTTCCT / CCAAGCGCCGGGATAGTTAT
85	Os02g0661100	ACGTTGGTGTCAGTAGGCTCAA / TGGCATCAAGCAGTCCATTG
86	Os10g0521900	GCCATGCTGTCGGAGCTT / AGAGCTGCAGCCTTGTTCGT
87	Os08g0384500	CAACCCGGCAACATGGA / AGCTGATCCCCAGTACGTCTTC
88	Os03g0218400	GCAATTTCGGCTCTGTACGTACT / CTCAAGAAGCACTCCGAATTGA
89	Os04g0461600	TTTTAGCTTCTGCTATATTTCATCTTGTC/CCAATGGAAACGCACACATC
90	Os02g0627100	CGGTGTTGTTTTTATCTGGTGAATA / CCGCTATGCAACGAAGAATTCT
91	Os03g0407900	CACCCCAGAAGGTCGTTAGG / GCATATGCCACACCCAAAACT
92	Os07g0502200	GATCACCGCACGAACCAAAT / CTGGCCTTCTTCTCCTGCAT
93	Os02g0562600	TGCTCGATTGGTCATAAGTGTCA / GCGTAGAGAGGGAGGGTGCTA
94	Os04g0634700	CGTTGATCAGGCACCATGTT / TTGGCCGCCACTTCTAGAGT
95	Os01g0882800	GCGTCGCCGAGGTCTTC / TGGAGAGCCAGGAGATCTGATC

96	Os07g0232800	CTCGCCGAGGACTTCATGA / CGAGCTGGAGCTTGCCTTT
97	Os09g0482800	TGCGTACTGTCCTCTTGCTGAA / CAAAATTTTCCATCCCGGATT
98	Os03g0773300	GGGAACAGCTCCGATGGA / GGCTTCGCTGGCTTGATC
99	Os01g0934400	GCGCAGATATAAACGCCACTCT / TCACATTGTTCGCTGGATCTTC
100	Os08g0138200	ACCTTCAACTCCGGCGATGA / GCAGATGAAGTACCGCGTGC
101	Os01g0690800	CCAACTAACCGCCCAACAAT / GACCGCTCGTGCTACCTTCT
102	Os03g0792800	CCAGGAGGCCGTATAAATCG / GCGAGATCCGCCATTCC
103	Os05g0541100	AAGCAAGGCAGAGCTGCAA / TGCCTCCAGATCGCTGTCT
104	Os01g0817000	TGTGTGGCACAAACCAAACTC / GTTAACGGACAACTCGTGAAGGT
105	Os01g0723800	TGACTTCTGGTATGGTGGGAAA / AAGACATCACCTGCGGAGATCT
106	Os07g0561800	GCCATTGGCGACATGGA / GTTGGAGGCGATCGATCTACA
107	Os01g0713200	CCCGTGATAGGCCAAACG / GGACACCACACGGCACTATG
108	Os02g0126400	AAGAGGACCTCCAAATCCACAA / CGACGTCTCATCTCGGTTCTTA
109	Os07g0119300	AAAGCAGCTCCCAGTCTGGAT / CCATGCCTGAGCCTGAGAAA
110	Os02g0682300	TGCTGTTAATCAACGGAGACAAA / AATCCTCGGCAGAAAAAGAAAA
111	Os07g0583600	CGTGGCACTAGCGTTGTGACT / CCAAGATGGTTACAAGGGAAGTTC
112	Os11g0184900	GAGCGAGGTTAAAAATAGTGGAGAA / CCATCCGGCAGAAACAAAAA
113	Os01g0570800	TTGGTTGCCAAAGATGGTGTT / TGGGAGGTAGTGACCGCTGTA
114	Os10g0466800	CAGCGGCAATGGGAGAGA / GGAATGGTCCGGGCTAAAG
115	Os04g0632100	CACACAGGCAAATCGACTGATC / GACCCTGCCTTTGGAGATGA
116	Os06g0288100	CAAGCGTGTCCACGGATTC / GACCCAACAAGTCCAGCACTAAG
117	Os02g0299300	CGAAGGACGCGCTAGCAT / GTGACCGAAAACGTGTGCTTT
118	Os07g0589000	CCTGCTCAATTCTCATATGCTTTC / GGCGAGAGGCTACCAAAGAGT
119	Os03g0285800	CACCGACCTCCATCACATCA / CTGGCAGTGCTCTTCTGACAGT
120	Os06g0292400	CCTGTGAGCATGGTATGAAGTTCTA / ATCAAGAGGCCAACCAATCCT
121	Os08g0492500	CCGATCGATGGAGAGGTGTAG / CCTTCTCATCTCTTCGCTTTCC
122	Os08g0386200	TCCTTTTGTTTCTCCCAACTCTCT / CCTCTCTTTTGCTTGACAGAAACC
123	Os02g0634800	AAGGCATGGCTGATTCATGT/CAGATGTTCACATGGTGACAGTA
